# Differentiation in neutral genes and a candidate gene in the pied flycatcher: using biological archives to track global climate change

**DOI:** 10.1002/ece3.855

**Published:** 2013-11-01

**Authors:** Kerstin Kuhn, Klaus Schwenk, Christiaan Both, David Canal, Ulf S Johansson, Steven van der Mije, Till Töpfer, Martin Päckert

**Affiliations:** 1 Biodiversity and Climate Research CentreSenckenberganlage 25, Frankfurt a. Main, D-60325, Germany; 2 Ecology and Evolution, Johann Wolfgang Goethe-UniversitätMax-von-Laue-Straße 13, Frankfurt am Main, D-60438, Germany; 3 Institute of Environmental Sciences, University of Koblenz-LandauFortstraße 7, Landau in der Pfalz, 76829, Germany; 4 Centre for Ecological and Evolutionary Studies (CEES), University of GroningenPO Box 11103, Groningen, 9700 CC, The Netherlands; 5 Department of Evolutionary Ecology, Estación Biológica de Doñana, CSICAv. Américo Vespucio s/n, Sevilla, 41092, Spain; 6 Department of Zoology, Swedish Museum of Natural HistoryPO Box 50007, Stockholm, SE 10405, Sweden; 7 Naturalis Biodiversity CenterPO Box 9517, Leiden, 2300 RA, The Netherlands; 8 Museum of Zoology, Senckenberg Natural History CollectionsKönigsbrücker Landstraße 159, Dresden, D-01109, Germany

**Keywords:** Avian *Clock* gene, biological archives, candidate genes, climate change, control region, microsatellites

## Abstract

Global climate change is one of the major driving forces for adaptive shifts in migration and breeding phenology and possibly impacts demographic changes if a species fails to adapt sufficiently. In Western Europe, pied flycatchers (*Ficedula hypoleuca*) have insufficiently adapted their breeding phenology to the ongoing advance of food peaks within their breeding area and consequently suffered local population declines. We address the question whether this population decline led to a loss of genetic variation, using two neutral marker sets (mitochondrial control region and microsatellites), and one potentially selectively non-neutral marker (avian *Clock* gene). We report temporal changes in genetic diversity in extant populations and biological archives over more than a century, using samples from sites differing in the extent of climate change. Comparing genetic differentiation over this period revealed that only the recent Dutch population, which underwent population declines, showed slightly lower genetic variation than the historic Dutch population. As that loss of variation was only moderate and not observed in all markers, current gene flow across Western and Central European populations might have compensated local loss of variation over the last decades. A comparison of genetic differentiation in neutral loci versus the *Clock* gene locus provided evidence for stabilizing selection. Furthermore, in all genetic markers, we found a greater genetic differentiation in space than in time. This pattern suggests that local adaptation or historic processes might have a stronger effect on the population structure and genetic variation in the pied flycatcher than recent global climate changes.

## Introduction

Global climate change has altered the phenology and distribution of many plant and animal species, resulting in mistiming such as disturbed ecological interactions across trophic levels (*i.e.,* predator prey or host parasite relationships) (review e. g., Parmesan 2006; Gienapp et al. 2008; Hansen et al. 2012). In particular, global warming is an important driving force for rapid adaptive changes of migration and breeding phenology in migrating birds (Cotton 2003; Gordo 2007; Both 2010; Gullett et al. 2013) as well as for local or regional demographic changes (Both et al. 2010). Stronger declines in population sizes of long-distance migrants have been observed in those populations that have suffered from climate-change-induced mistiming, as the synchrony between the birds and their food (e.g., bird laying dates and caterpillar abundances) has been distorted (Visser and Both 2005). However, such population declines are more pronounced in Western Europe, where the rate of spring warming is higher than in northern Europe (Visser et al. 2003; Both et al. 2004, 2010).

**Figure 1:**
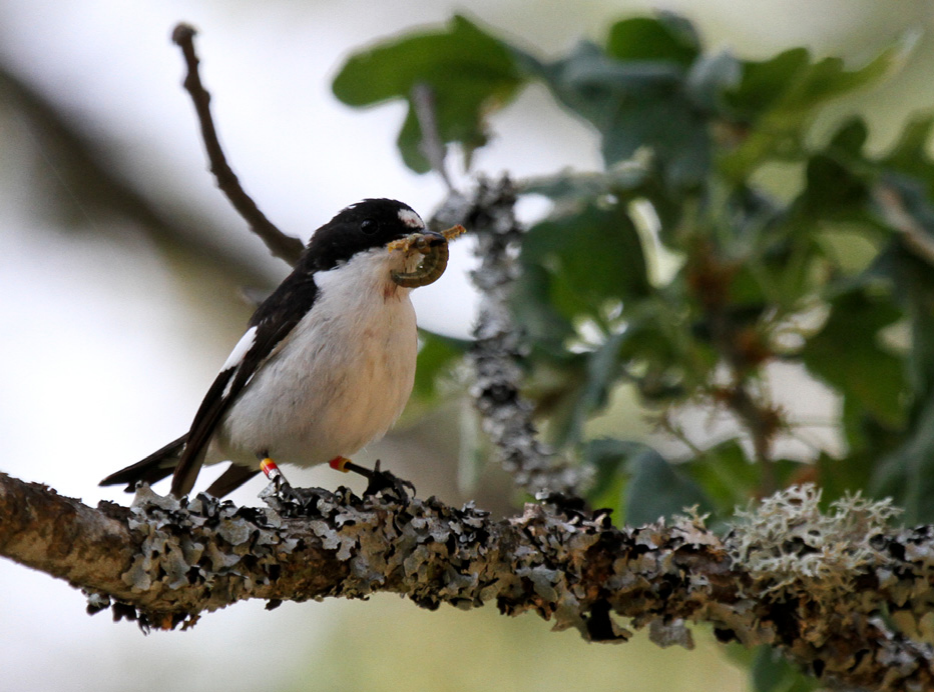
Pied flycatcher (*Ficedula hypoleuca*), male carrying caterpillar as nestling food (photo: Carlos Gutiérrez-Espósito). During recent decades, western European populations have strongly declined due to climate-induced changes to earlier food peaks in oak forest habitats.

The pied flycatcher (*Ficedula hypoleuca*) is a long-distance migrating passerine that has undergone remarkable changes in life-history traits in response to climate change (Fig. 1). Over the last two decades, this species has shifted toward earlier laying dates in response to increased local temperatures (Both et al. 2004; Laaksonen et al. 2006) particularly in local Dutch populations with early caterpillar food peaks (Both and Visser 2005). Despite the advancement in laying dates, this temporal shift has been insufficient to compensate costs associated with the earlier development of prey species. In areas with a late food peak, there was only a weak population decline of about 10%, while in habitats with an early food peak, such as oak forests, populations declined up to 90% (Both et al. 2006). These dramatic regional changes in population sizes might have a measurable effect on genetic diversity over time. To date, the manifold studies on the pied flycatcher genome were mainly concerned with differential selective pressures on autosomal and z-linked markers (Sætre et al. 2001; Ellegren et al. 2012; Hogner et al. 2012; Backström et al.^2013^) and on candidate genes encoding for plumage coloration (Lehtonen et al. 2012), while the timely aspect of very recent demographic changes was rather neglected. In order to document such changes, it is possible to utilize biological archives, such as museum collections, for genetic analyses if no long-term data sets are available (Wandeler et al. 2007; Ramakrishnan and Hadly 2009). In a number of seminal studies, museum collections have been used to document, for example, the loss of genetic diversity in endangered species, the level of anthropogenic influences on population sizes and connectivity, range shifts, cryptic introductions as well as genetic introgression (e.g., Nystrom et al. 2006; Hekkala et al. 2011; Mende and Hundsdörfer 2013). However, most of these studies have focused on temporal changes in genetic variation in neutral genetic markers, whereas only a few have examined the genetic variation in candidate genes (e.g., Hartley et al. 2006). Although recent environmental and global climate changes are in the focus of current biodiversity and evolutionary research (Reusch and Wood 2007), only a few studies have compared individuals from museum collections and current populations to assess the genetic response of populations to temperature (Hadly et al. 2004; Balanya et al. 2006; Nielsen and Bekkevold 2012).

Here, we studied variation in selectively neutral mitochondrial and nuclear loci as well as one candidate gene in several current and historic European pied flycatcher populations, to test for potential effects of global climate change on the genetic architecture of populations. Sampling sites covered a large part of the species' breeding range, from Spain in the south to Germany, the Netherlands, and to Scandinavia in the north. First, we estimated the genetic variation in recent populations, and secondly, we compared current and historic samples, expecting stronger changes of genetic diversity over time in Dutch populations which underwent a more steep decline (Both et al. 2010) than the more stable populations of Eastern Germany and Sweden (Steffens et al. 1998; Flade and Schwarz 2004).

Our third aim was to unravel patterns of natural selection caused by climate change. To this end, we also considered non-neutral genetic variation in a candidate gene approach. Based on the assumption that genes which are known to mediate behavior in one species are likely to mediate comparable behaviors in other species (Fitzpatrick et al. 2005), we have chosen a putative non-neutral candidate locus related to temperature-dependent breeding behavior. The *Clock* gene locus belongs to a group of regulatory genes of the circadian clock in a wide range of invertebrate and vertebrate organisms (Yoshimura et al. 2000; Helfer et al. 2006; Zanquetta et al. 2010). Variation in *Clock* genotypes is characterized by allele length polymorphism in the poly-Q region and was shown to change gradually along latitudinal clines in vertebrate model species (O'Malley and Banks 2008; O'Malley 2010). Also in some passerine birds, *Clock* gene poly-Q length variation is associated with latitude of breeding site (Johnsen et al. 2007), breeding phenology (Liedvogel et al. 2009; Caprioli et al. 2012) and even with fitness-related traits such as clutch size (Dor et al. 2012) or timing and distance of juvenile dispersal (Charkarov et al. 2013). Despite of some counterevidence of such adaptive variation in other bird species (Liedvogel and Sheldon 2010; Dor et al. 2011), the *Clock* gene locus is currently suggested to be a component of a “migratory gene package” that controls physiological adaptations associated with a number of migratory behavioral traits (Liedvogel et al. 2011).

We therefore expected that the *Clock* gene underlies natural selection in our model organism particularly in those populations which are subjected to strong environmental or demographic changes driven by global warming. Specifically, we expected (i) geographical differences and (ii) differences over time in populations where breeding phenology has changed but less differentiation over time where breeding phenology did not change.

Fourthly, if global climate change had a significant effect on natural populations of pied flycatchers, we expected genetic differences over time to be as large as, or even larger than those among local populations (within a given time period). Directional selection may cause the overall loss of genetic variation, resulting in fewer haplotypes and alleles or other indices of molecular diversity. An even stronger effect is expected (given that populations are reasonably isolated) in fitness-related genes, such as genes responding to environmental cues determined directly (temperature) or indirectly (food availability) by global climate changes, for example, the *Clock* gene. Natural selection may alter gene frequencies even in short time periods, resulting in higher genetic differentiation at candidate loci than at neutral loci. In this study, we tested this hypothesis by comparing spatial and temporal differentiation in mitochondrial DNA, microsatellite loci, and a candidate gene (*Clock* gene).

## Materials and Methods

### Sampling

**Figure 2:**
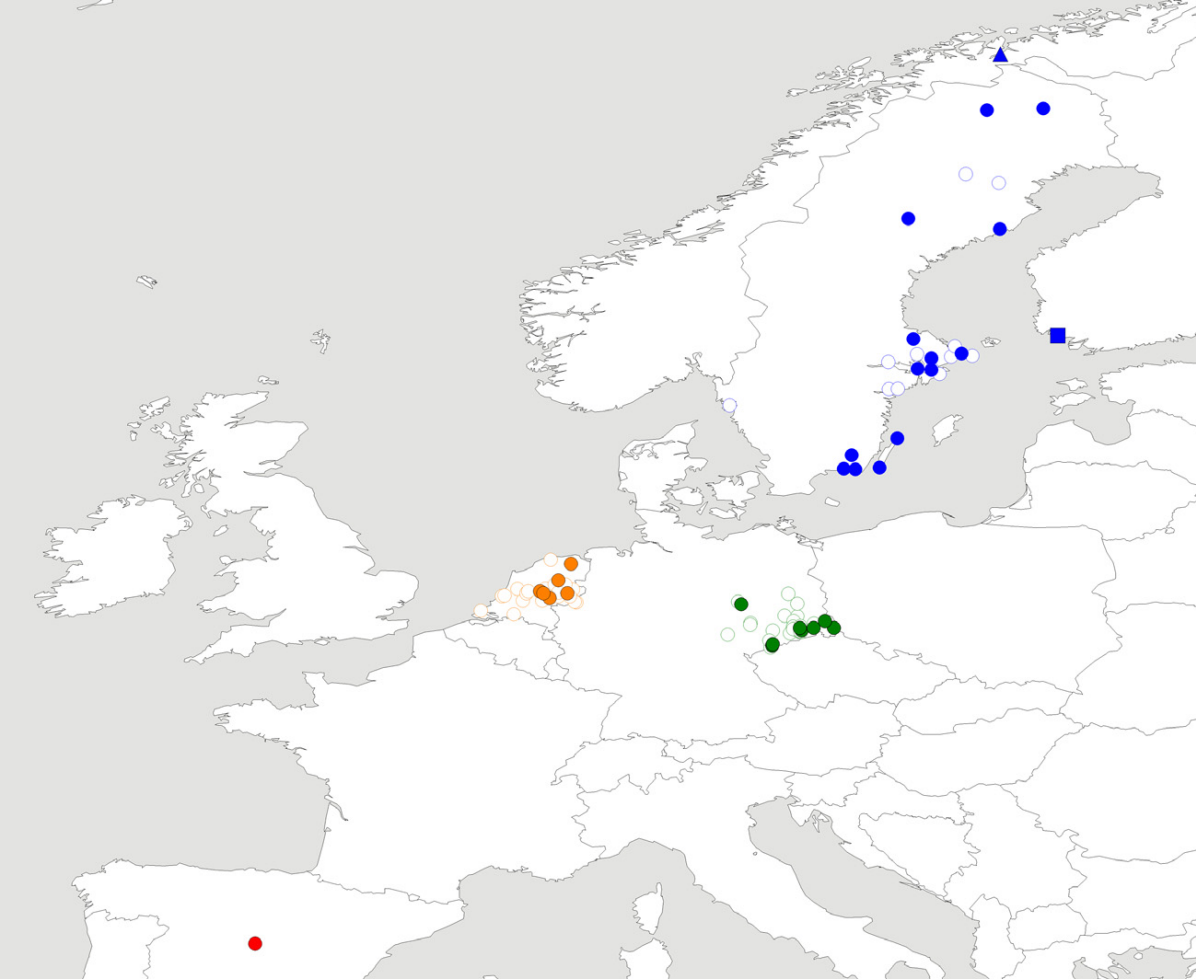
Origin of *Ficedula hypoleuca* samples used for population genetic analysis across Europe. Filled colored symbols denote samples from extant populations (1995 and subsequent), and open colored symbols denote historic samples (1977 and previous).

We investigated populations of pied flycatchers from six regions in Europe: Finland, Germany, Norway, Spain, Sweden, and the Netherlands. Altogether, 311 samples were collected from specimens dating between 1901 and 2010 (for origin of samples and collection dates see Fig. 2, Table S1). Most samples collected before 1990s were taken from nest box populations or tissue collections, while specimens collected before the 1980s were sampled from study skins or feather collections of various museums. We chose only samples collected during the regional-specific breeding periods (Cramp and Perrins 1993; Clement and Taylor 2006; Newton 2008) to ensure sampling of local populations. This resulted in *a priori* limited availability of appropriate specimens in all collections. Samples taken in the field (1990s and later) comprised blood and tissue (both stored as frozen material) and feather samples (stored dry or in alcohol; see Table S1). Feathers were always sampled at the calami. From historic collection material (whole skins), we usually took toe pad tissue for DNA extraction, but we also took a small skin patch from the ventral side of some historic specimens (and in very few cases, feathers from mounted feather collections; Table S1).

### DNA isolation

DNA from frozen tissue and blood samples, as well as from nestling feather samples kept in alcohol (recent material), was extracted using the DTAB method (Gustinich et al. 1991). Extraction from toe pads and skin patches (historic museum material) was carried out in a separate clean laboratory. There each step of analysis (sampling and extraction) was carried out on separate working benches. DNA from museum specimens and dry feather samples from extant populations was extracted using the sbeadex® forensic kit (LGC Genomics, Berlin, Germany) according to the manufacturer's instructions with small modifications, for example, overnight incubation of tissue with proteinase K (instead of one hour). In order to yield a sufficiently high DNA concentration for microsatellite analyses, we performed two successive elution steps with a volume of 20 μL of sterile water (instead of one step with 60 μL elution volume). In order to avoid cross-contamination, extant and historic samples were never treated at the same time, and after each step, working benches were cleaned with DNA-away (Molecular Bio Products, Inc., Toronto, ON, Canada), and both benches and laboratory rooms were set under UV-light for at least four hours. The amount and the quality of DNA isolates were checked on 1.4% agarose gels.

### PCR amplifications

Control region: We successfully amplified a 271-bp fragment of the mitochondrial control region of 309 pied flycatcher individuals. Primers were designed on the basis of an alignment of control region sequences of different bird species belonging to the family Muscicapidae. We used the forward primer crFHfor 5′GCCATACGGGTATGCTATG 3′ and the reverse primer crFHrev 5′GCTTGGGAGTTCCTGAAAG 3′. PCRs were performed in 12.5 μL using a Tetrad PTC-225 thermal cycler (BIO-RAD, München, Germany). Each reaction contained about 1–10 ng template DNA, 2.5 pmol each primer, 0.2 mmol/L each dNTP, 2 mmol/L MgCl2, 1× amplification buffer, and 0.5 U Taq DNA polymerase (Invitrogen, Darmstadt, Germany). The PCR started with an initial denaturation at 94°C for 1 min followed by five cycles: 94°C 40 sec, 45°C 40 sec, and 72°C 1 min; then cycling 35 times: 94°C 40 sec, 52°C 40 sec, 72°C 1 min; ending with a final extension at 72°C for 5 min. PCR products were separated by electrophoresis using 1.4% agarose gels and visualized by staining with ethidium bromide (1 ppm). In agreement with Töpfer et al. (2011), historic DNA isolates from foot pads yielded better results than DNA isolates from skin patches. After purifying the PCR products with Agencourt AMPURE (Beckman Coulter), both strands were sequenced with the following PCR program: 96°C 20 sec, 50°C 20 sec, 60°C 4 min, and 39 cycles, using GenomeLab DTCS-Quick Start Kit (Beckmann Coulter, Krefeld, Germany). Dye terminators were removed with the Agencourt CleanSEQ kit (Beckmann Coulter), and amplicons were analyzed on a CEQ 2000 (Beckman Coulter) automatic sequencer.

From the first DNA extracts, all historic samples were amplified and sequenced at least twice. In all cases, both sequencing reactions yielded identical results. In order to control for the possible occurrence of single-base errors in PCR products from museum specimens (Sefc et al. 2007), DNA was newly extracted from a set of 126 original samples and was re-analyzed via independent PCR and sequencing reactions (most of these were historic samples plus a few recent samples that carried unique haplotypes). Each step of analysis (sampling, extraction, and PCR) was performed in a clean laboratory on separate working benches that were decontaminated by UV-light exposure after each working step. Negative controls were performed for both DNA extractions and PCRs in the control analysis, and spot tests of PCRs from negative controls were also sequenced in order to test for possible contamination. Sequences of negative controls never showed a considerable signal. For haplotypes that deviated from those resulting from the first analysis, the respective samples were re-analyzed at least one more time (starting from PCR). For 14 samples, haplotypes from the first analysis were not reproducible in the control analysis (11%). However, all of these deviating haplotypes were reproducible in one or even two replicate PCR and sequencing reactions from the second DNA extract. For seven of fourteen ambiguous samples, deviations between first and control sequencing were due to double peaks at the respective polymorphic sites. Among the ten polymorphic sites concerned, six cases corresponded to the more common overlaid signals of either the two purine or the two pyrimidine bases (C + T or A + G double peaks).

#### Microsatellites

Ten microsatellite loci were amplified using primers published by Leder et al. (2008). We chose primer combinations with nonoverlapping product sizes, using two different color labels, and tested them for the feasibility of multiplexing. As we use historic samples, we selected product sizes with a maximum length of around 400 bp. End-labeled forward primers were obtained with either Alexa647 (Invitrogen) or IRD700 (Metabion, Martinsried, Germany) fluorescent dye (see Table S2). Multiplex PCRs were performed with the Type-it Microsatellite PCR Kit (QIAGEN, Hilden, Germany) according to instructions of the manufacturer. The PCRs were performed in a S1000 thermocycler (BIO-RAD), starting with an initial denaturation at 95°C for 3 min, then 32 cycles of 95°C 30 sec, 55°C 90 sec, 72°C 60 sec, and a final extension of 60°C for 30 min. All historic samples were analyzed at least two times independently (see above; Morin et al. 2001). Microsatellite loci longer than 200 bp were amplified separately for all museum samples, using the same PCR program and final primer concentrations of 0.2 μmol/L each. PCR products (1 μL) were directly mixed with SLS and a size standard (DNA Size Standard Kit-400 Beckmann Coulter), electrophoresed and analyzed on a CEQ 2000 (Beckman Coulter) automatic DNA sequencer.

#### *Clock* gene

For initial PCR tests, we used the primer pairs from Johnsen et al. (2007), to amplify the variable region of the *Clock* gene locus (corresponding to the human *Clock* gene exon 20; Steeves et al. 1999) with the following settings: initial denaturation at 94°C for 2 min, then 35 cycles of 94°C for 30 sec, 58°C for 30 sec, 72°C for 1 min and a final extension at 72°C for 7 min. In order to verify PCR products, a subset of 37 fragments was sequenced from Dutch and Spanish samples with 25 cycles of 96°C for 10 sec, 50°C for 5 sec, and 60°C for 4 min. Finally, the *Clock* gene locus of pied flycatchers was screened for length polymorphism by PCR amplification and electrophoresis using an automatic DNA sequencer. PCR was conducted with an Alexa647 labeled forward primer 5′TGGAGCGGTAATGGTACCAAGTA 3′ and the reverse primer 5′ TCAGCTGTGACTGAGCTGGCT 3′ (Johnsen et al. 2007) with a final concentration of 0.2 μM each. We applied the same PCR conditions and detection methods as described for the microsatellites, except that PCR products from recent samples were diluted 1:10 to 1:100 before electrophoresis.

Museum samples for which we could not perform at least two replicates with concordant results for each marker (control region, microsatellites, and *Clock* gene), or which were genotyped with less than eight of ten microsatellites, were discarded from further analyses. Swedish pied flycatchers collected on Öland are likely to be migrants heading to northern Sweden (Fransson and Hall-Karlsson 2008). As we could not rule out that they also may migrate to Finland or Norway, we reanalyzed our data excluding these specimens. The exclusion of all potential migrants from Swedish populations did not alter the main picture, and therefore, we report results based on the complete data set.

### Statistical analysis

Samples were assigned to groups according to their geographical origin. Dutch, German, and Swedish samples were subdivided into extant and historic samples and Spanish, Finish, and Norwegian samples were classified as three further recent populations only, because no historic sampling was available from these regions. On the temporal scale, we had a considerable sampling gap in the 1980s (from 1977 to 1995), and therefore, we labeled all samples collected before 1977 as “historic” or “museum samples” (*N* = 110) and those samples taken after 1995 as “extant” or “recent” (*N* = 201). This temporal division coincides with the first observations of decline in pied flycatcher populations in the Netherlands in the mid-1980s, as well as with a general decline of forest-dwelling long-distance migrants during the same time (Both et al. 2010).

Control region sequences as well as *Clock* gene sequences were aligned with BioEdit 7.0.9.0 (Hall 1999) and verified by eye. Nucleotide and haplotype diversities for the control region were calculated with the program DnaSP 5.10 (Librado and Rozas 2009). The allelic richness for microsatellite loci and unbiased estimators of *F*_st_ (e.g., G′′_ST_) for microsatellite loci and the *Clock* gene and Φ_ST_ for mtDNA were evaluated with GenAlEx ver. 6.5 (Meirmans and Hedrick 2011; Peakall and Smouse 2012). Private haplotypes, private alleles, observed heterozygosity (*H*_o_) for microsatellite loci and the *Clock* gene were estimated using Arlequin 3.5.1.2 (Excoffier and Lischer 2010). Genetic differentiation among populations (*F*_st_) for neutral and non-neutral markers was also calculated with Arlequin 3.5.1.2. (Excoffier and Lischer 2010).

The ten microsatellite loci were tested with MICRO-CHECKER 2.2.3 (van Oosterhout et al. 2004), using 1000 randomizations and a 95% confidence interval, for genotyping errors due to stuttering, large allelic dropout, and null alleles.

In order to investigate the phylogeographic relationships among haplotypes, we constructed networks with the program Network 4.6.0.0. (Bandelt et al. 1999; Forster et al. 2001; Polzin and Daneshmand 2003). The median-joining method with transversions weighted three times more than transitions was used to build a network. Both the network and the alignment were cross-checked for any kind of sequencing artifacts, particularly with respect to the historic sampling. Amplification errors can be detected by an increased frequency of unique tip haplotypes (occurring in single individuals) mostly characterized by C to T substitutions in the network representing historic museum specimens (Stiller et al. 2006; Sefc et al. 2007). For graphical illustration, we chose the “frequency one criterion”, which excludes unique haplotypes, and the star contraction algorithm in order to simplify the network.

The model-based clustering method structure by Prichard et al. (2000) was used to infer the number of clusters (K) of individuals based on microsatellite data. Simulations were run assuming the admixture model with correlated allele frequencies and using the sampling location as prior. Ten independent runs, with ten replicates for each K (1–10), were performed with a 500,000 burn-in period followed by 500,000 MCMC repeats after burn-in. To find the true value of K, we performed the K-statistic (Prichard et al. 2000). The results were compared with K estimates based on the method by Evanno et al. (2005) as implemented in STRUCTURE HARVESTER (Earl and vonHoldt 2011).

In order to analyze the relationship between poly-Q genotypic variation and geographical origin, gender, and laying date (General linear model (GLM) in STATISTICA (2010)), we defined poly-Q genotypes as the sum of the poly-Q repeats of both alleles, as it has been shown that females (unlike males) with fewer Q repeats tend to breed earlier in the season (Liedvogel et al. 2009). Associations between pairwise population differentiations (G′′_ST_) based on mitochondrial and microsatellite loci as well as microsatellite loci and the *Clock* gene locus were tested using a Mantel test (Peakall and Smouse 2006; GENALEX version 6 and 6.5).

## Results

Nearly all frozen blood and tissue samples (collected after 1980) were successfully used for the amplification and sequencing of the control region, *Clock* gene and for microsatellite analysis (Table S1). For the period of 1940–1979, control region sequences were recovered in 98% of all samples and at least eight microsatellite loci (which were included in the analysis) were successfully amplified in 74% of all cases. Samples collected before 1940 yielded good sequencing results with regard to mtDNA (91%), but for most samples older than 1940, less than ten microsatellite loci were amplified, mostly due to dropout of some loci of a length greater than 320 bp (only 9% of the samples from this period scored eight or more loci; see Table S1). Regardless of locus length, the success rate of PCR amplifications from samples older than 34 years predominantly depended on the quality but not on the age of the preserved specimens.

### Neutral genetic differentiation

Control region: A total of 304 pied flycatcher individuals from nine populations (Table 1) were analyzed for variation in a 271-bp fragment of the mtDNA control region. Among all individuals examined, a total of 61 haplotypes were identified (27 variable sites), ranging from eight in the current Swedish population to 20 in the current as well as in the historic German population. The most common haplotype was found in 59 individuals (almost 20%) and was widely distributed across all extant and historic populations except those from the Iberian Peninsula. Three other common haplotypes were present in 39, 36, and 25 individuals, respectively, and had similar geographical and temporal distributions. About two-thirds (65.6%) of all haplotypes represented private haplotypes. The historic and the recent German population harbored 40% private haplotypes, whereas the current and historic Dutch, the Finnish, the Norwegian, and the current and historic Swedish populations shared more than 70% (73–82%) of their haplotypes with other populations. Only the Spanish population showed a highly distinct pattern, that is, nine private haplotypes but only one haplotype shared with other populations. Nucleotide diversities were largely similar among geographical and temporal samplings and ranged from 0.006 to 0.0095 (with highest values in the extant Norwegian and the historic Swedish population; Table 1). There was no considerable difference of nucleotide diversities among the temporal samples from the same region (Germany, Sweden, and the Netherlands, Table 1).

**Table 1:** Summary statistics and population genetic parameters of recent and historic *Ficedula hypoleuca* populations.

	Control region				Microsatellites				*Clock* gene			
			
	*n*	*h*	ph	π	*n*	*a*	pa	*H*_o_	*n*	*a*	pa	*H*_o_
Recent												
Finland	16	10	2	0.00852	16	78	2	0.681	16	4	0	0.563
Germany	38	20	8	0.00833	36	100	5	0.700	32	4	0	0.531
Norway	16	11	2	0.00923	16	83	3	0.719	16	3	0	0.438
Spain	45	10	9	0.00745	45	99	15	0.722	43	4	0	0.535
Sweden	18	8	2	0.00794	18	89	8	0.783	17	3	0	0.412
The Netherlands	67	17	4	0.00834	68	124	14	0.737	66	5	0	0.500
Historic												
Germany	53	20	8	0.00855	32	90	6	0.734	35	5	0	0.457
Sweden	20	11	2	0.00948	18	82	4	0.733	20	4	0	0.500
The Netherlands	31	14	3	0.00595	24	102	12	0.697	30	4	0	0.400
Total	304	61*	40		273	208	69		275	5	0	

*n*, number of individuals analyzed; control region: *h*, number of haplotypes; ph, number of private haplotypes; π, nucleotide diversity; microsatellites and *Clock* gene; *a*, number of alleles; pa number of private alleles; *H*_o_, observed heterozygosity.

* Total number of haplotypes, rows do not sum up because haplotypes are shared among populations.

The overall Fixation index for genetic differentiation between all populations was 0.26, and thus, about 10-fold higher than that obtained for the microsatellites (see below). 73.72% of the total variation was found within populations. Pairwise *F*_st_ estimates varied from zero (e.g., among Scandinavian populations) to about 0.5 for all pairwise comparisons with the Spanish population (Table S3). Significant differences were estimated for 56.5% of all pairwise comparisons. All Scandinavian populations, as well as historic and the recent populations from Germany, Sweden, and the Netherlands, were not significantly differentiated from each other.

#### Microsatellites

A total of 273 individuals from nine populations were successfully screened for variation at ten microsatellite loci. Only a few populations–marker combinations showed homozygote excess (17 of 90) or stuttering (2 of 90), and no evidence for large allelic dropout (12 of 90) was found. Private alleles were found in all populations (Table 1). The Spanish (15.15%) and the historic (11.76%) and recent (11.29%) Dutch populations exhibited the highest percentage of private alleles, whereas the lowest proportion was found in the Finnish and Norwegian populations (2.56% and 3.61%, respectively). The observed heterozygosity (*H*_o_) covered a range from 0.681 to 0.783 with a mean of 0.722 (Table 1).

**Figure 3:**
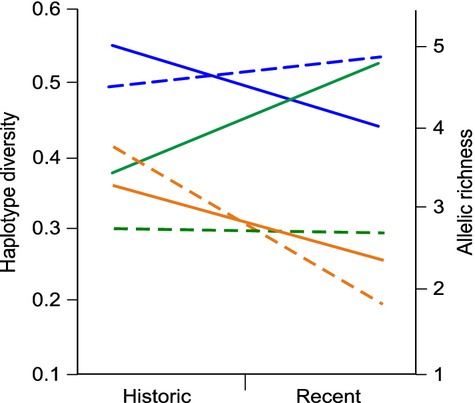
Changes over time in haplotype diversity (number of haplotypes/*N*; lines) and in allelic richness (number of alleles/*N*; dotted lines) for *Ficedula hypoleuca* populations. Dutch populations are marked in orange, German in green, and Swedish in blue.

The overall fixation index was 0.02 and more than 10-fold lower than mitochondrial differentiation. 97.91% of the variation was found within populations. Pairwise comparisons between populations ranged from zero (recent Germany/Norway) to 0.05 (Spain/historic Netherlands). In concordance with control region divergence, we found that the Spanish population was significantly differentiated from all other populations at nuclear loci (Table S3). In contrast to the mtDNA results, which revealed no significant differentiation among Scandinavian populations, pairwise F-statistics based on microsatellite data indicated a low but significant level of genetic differentiation between the Finnish population and both the recent and the historic population from Sweden. When historic and recent populations from the same country were compared, only the German populations showed a little, but significant differentiation (*F*_ST_ = 0.008; *P* = 0.018 ± 0.012). Overall, we observed a tendency of less genetic variation (haplotype diversity and allelic richness) in the current Dutch population than in the historic Dutch population (Fig. 3).

### Population structure and phylogeographical patterns

In the control region alignment, we did not find any bias with respect to an increased frequency of C to T substitutions in the historic sampling. The network based on recent and historic samples (61 haplotypes) contained nine unique tip haplotypes (not shown); however, among these, we found no bias with respect to our historic sampling. In fact, eight unique tip haplotypes occurred in the extant populations.

The network based on recent samples only (Figure S1) displayed a strong genetic differentiation of the Spanish population, that is, sharing only one rare haplotype (which occurs only once in each population) with the Finnish and Swedish populations. All other haplotypes were exclusively found in the Spanish population. There was no geographical structure observed among the other European populations. The majority of haplotypes was shared at least between two populations, and three common haplotypes were found in all populations except Spain.

**Figure 4:**
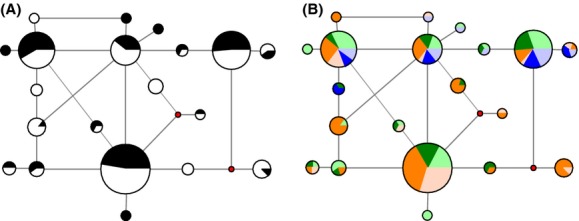
Median-joining haplotype networks for recent and historic *Ficedula hypoleuca* populations, based on mtDNA control region sequences. (A) Overview of recent (white) and historic (black) haplotypes found in Germany, Sweden, and the Netherlands. (B) Haplotypes are additionally color coded according to their geographical and temporal origin (Germany: green; Netherlands: orange; Sweden: blue; historic samples: light color). Only haplotypes which occur more than once are shown. The size of the circles is proportional to the frequency of the haplotypes, and the lines indicate single mutation. Small red dots denote median vectors.

In order to describe the distribution of haplotypes among current and historic populations, we reconstructed a network using control region sequences of Dutch, German, and Swedish populations. Like the other analyses (microsatellites and candidate genes), this revealed very little temporal differentiation (Fig. 4A) and again no geographical differentiation (Fig. 4B). Nearly all haplotypes of the network were represented in both recent and historic samples. Only four haplotypes were exclusively found in recent or historic populations, respectively. Dutch and German populations shared 7 haplotypes which did not occur in the Swedish population.

Employing a mode-based clustering method for microsatellite data of all recent samples, individuals were probabilistically assigned into K = 4 populations (Figure S2). Individuals from Spain clearly formed a cluster of their own. The second cluster corresponded to birds from Sweden, and the third exhibited samples from Finland, Germany, and Norway. The fourth cluster harbored all individuals from the Netherlands. In Structure analyses which resulted in K = 3, the Swedish populations fell into the cluster with Finland, Germany, and Norway. Using no prior information on sample geographical origin, K = 2 was identified as the most likely. Only the Spanish birds could be discriminated from all other populations. This pattern was supported by Evanno′s ΔK method.

### Non-neutral genetic differentiation

Altogether, 275 pied flycatchers, belonging to nine different populations (geographical and temporal), were genotyped for length variation at the poly-Q locus of the *Clock* gene. We identified five length variants ranging from 193 bp (10 poly-Q repeats) to 205 bp (14 poly-Q repeats). At least three different alleles were found in each population, and all five alleles were detected in the current Dutch as well as in the historic German population. No pied flycatcher population exhibited private alleles. However, allele 205 was only found once in a historic German sample and in one recent sample from the Netherlands, and allele 193 was not found in extant Norwegian and Swedish samples. The remaining alleles were found in all populations with allele 199 being much more abundant in all populations than the other two. The observed heterozygosity (*H*_o_) was consistent over the populations and ranged from 0.400 to 0.563 (Table 1).

Pairwise comparisons among all populations yielded *F*_st_-values ranging from zero to 0.0174. Only the pairwise comparison between the recent populations from the Netherlands and Spain showed low (*F*_ST_-value = 0.0174), but significant differentiation at the *Clock* gene locus (Table S3). The overall genetic differentiation index F_st_ was zero. Results from AMOVA highlight that no variation was found among populations, but 100% of the variation is explained by variation within the populations.

#### Poly-Q variation: Sex bias

A subset of 205 samples was further analyzed for sex-specific differentiation at the poly-Q locus. GLM analysis performed in STATISTICA yielded no significant differences between geographical regions (*P* = 0.54) nor any interaction of population x gender (Table S4). Forty-two pied flycatcher individuals from the Spanish nest box population were tested for a correlation between laying dates (males were assigned the laying date of their mated female) and poly-Q genotype variation. We did not find any association of the two parameters for either females (*N* = 26; *r* = −0.022) or males (*N* = 16; *r* = 0.051).

### Comparison of candidate genes with neutral markers

**Figure 5:**
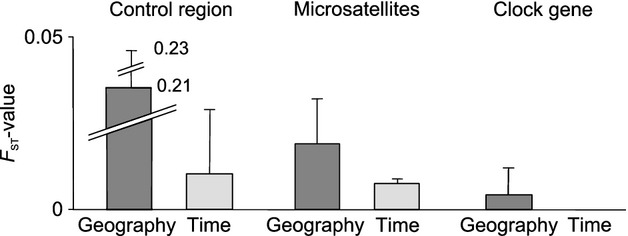
*F*_st_-values and standard deviations for pairwise comparisons of control region sequences, microsatellite loci, and *Clock* gene loci, between recent samples from Finland, Germany, Norway, Spain, Sweden, and the Netherlands (geography) and pairwise comparisons between recent and historic samples from Germany, from Sweden, and from the Netherlands, respectively (time).

**Figure 6:**
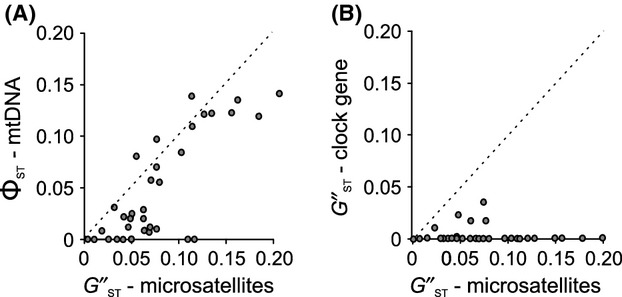
Pairwise Φ_st_ (mtDNA) and G′′_ST_ (microsatellites and *Clock* gene) comparisons between all populations. Dotted line in each graph (5A and B) indicates the 1:1 neutrality expectation of similar differentiation among both genetic markers. (A) is based on microsatellite loci and control region sequences and (B) is based on differentiation in microsatellites and *Clock* gene alleles. The same patterns are observed if only recent populations were analyzed (data not shown).

Regardless of the marker system used, geographical differentiation was much more pronounced than differentiation between historic and recent samples from the same country (Fig. 5). This was also the case if the highly differentiated Spanish population was excluded from the analysis (data not shown). Genetic differentiation measured by *F*_st_-values was about 10-fold higher on mtDNA level (control region) compared with nuclear markers (microsatellites and *Clock* gene). However, there was a significant association between pairwise Φ_st_ values based on mtDNA and G′′_ST_ values based on microsatellite loci (*r*^2^ = 0.64; Mantel test, *P* = 0.01; Fig. 6A). On the other hand, G′′_ST_ values based on microsatellite loci were not significantly correlated with G′′_ST_ values based on *Clock* gene loci (Mantel test, *P* = 0.38; Fig. 6B). In contrast, differentiation at the *Clock* gene locus was significantly lower than differentiation at neutral loci (*t* = 8.2705, df = 36.672, *P* < 0.001, Welch two sample *t*-test) which is in concordance with the finding that populations showed hardly any genetic divergence at the *Clock* gene locus.

## Discussion

Contrary to our expectations, genetic differences on the temporal scale within populations were much weaker than spatial differentiation, and only a slight temporal decline of genetic variation was found in the Dutch population. Thus, any postulated negative effect of climate change on local genetic variation, particularly in declining Western European populations, would have been counteracted or mitigated by gene flow. Second, despite very little genetic variation in the *Clock* gene locus among our study populations, the overall pattern of greater genetic differentiation on the spatial scale compared with the temporal scale was similar at this candidate gene and neutral loci.

### Spatial genetic differentiation in neutral markers

In general, the low degree of genetic population structuring of bird species (Crochet 2000) mainly originates from historic processes, in particular cases of rapid range expansions (in the pied flycatcher: Backström et al. 2013; in tits: Kvist et al. 2002, 2003). Spatial differentiation patterns driven by dispersal strongly contrast to those driven by vicariance (for birds see Voelker 1999; Zink et al. 2000). The extant distribution of western Palearctic *Ficedula* flycatcher species has been largely shaped by historic processes such as Pleistocene range fragmentation (for significant East-West subdivision of other Eurasian bird species see Haring et al. 2007, 2012; Zink et al. 2008). Their present distribution includes secondary overlap and hybrid zones (Sætre et al. 2001; Hogner et al. 2012) as well as significant population subdivision in pied flycatchers with respect to Iberian populations (this study; Lehtonen et al. 2009, 2012).

The geographical differentiation among extant European pied flycatcher populations in mitochondrial DNA (*F*_st_ = 0.34) and nuclear loci matches well the findings by Haavie et al. (2000). The significant differentiation of populations from the Iberian Peninsula versus the Central and Northern European populations is strongly corroborated by both neutral marker systems (compare also Haavie et al. 2000; Lehtonen et al. 2009; however, no confirmation of that pattern from an autosomal marker set in Backström et al. 2013). Similar phylogeographic patterns have been described for other species, such as Iberian and central/northern European chiffchaffs, *Phylloscopus ibericus* and *P. collybita* (Helbig et al. 1996, 2001) and green woodpeckers *Picus viridis* (Pons et al. 2011). Even within the northwestern breeding range of the pied flycatcher shared haplotypes and alleles as well as *F*_st_ statistics suggest some minor but not unequivocal differentiation of the Dutch and German populations against those from Scandinavia. These findings are in concordance with results of Lehtonen et al. (2009).

The large discrepancies in intraspecific variation between nuclear and mitochondrial markers (such as a 10-fold lower fixation index of microsatellite alleles compared with mtDNA haplotypes) could be related to differences in rate and patterns of mutations (Haavie et al. 2000; Ballard and Rand 2005). This discrepancy has also been documented for Swedish pied flycatcher populations (Tegelström et al. 1990) and might be explained by gender-biased gene flow and dispersal (Prugnolle and de Meeus 2002; Hedrick 2007). The slight geographical structure in mtDNA across Central Europe would suggest female-biased dispersal, which is consistent with the general observation that in passerines (including pied flycatchers), dispersal is female-biased (Clarke et al.^1997^; Winkel and Hudde 2001; Eeva et al. 2008). However, long-distance dispersal of pied flycatchers between the UK and the Netherlands seemed to be biased more by males than females (Both et al. 2012), and these relatively rare long-distance dispersal events could profoundly affect genetic structure.

### Temporal genetic differentiation in neutral markers

Besides processes such as historic gene flow and succession of new populations and subsequent founder effects, also recent demographic changes might have altered the genetic architecture of pied flycatchers. Recent population declines due to climate change, mainly in Dutch populations (Both et al. 2006), raised the question whether genetic drift might have reduced local genetic diversity. Loss of genetic diversity over time, in marginal or small isolated populations, for example, on islands, has been documented in a few studies, for example, in the Spanish White-headed Duck, *Oxyura leucocephala* (Muñoz-Fuentes et al. 2005), the Floreana Mockingbird, *Mimus trifasciatus*, on two remote Galapagos islands (Hoeck et al. 2011) as well as in two continental drastically bottlenecked species, the North American whooping crane, *Grus americana* (Glenn et al. 1999), and the European bearded vulture, *Gypaetus barbatus* (Godoy et al. 2004). Our comparison of population genetic variation suggests that the demographic declines in Dutch populations have resulted in a decrease in haplotype diversity and allelic richness over time and demonstrated that the current Dutch populations exhibited the lowest levels of diversity of all European populations. These patterns are consistent with the expectation that global climate change may have a weaker or no effect on the genetic composition of Northern European pied flycatcher populations, because spring phenology has not advanced here as strongly as in Western Europe (Both et al. 2004). However, the decline in genetic variation in the Dutch population was not consistent in all parameters (see Table 1; e.g., π). Firstly, in the Netherlands, local population declines differed regionally and largely in dimension (in oak stands up to 90% decline: Both et al. 2006). Although our sampling involved Dutch populations that were less affected by climate-induced demographic changes (12–20%), the overall regional diversity could still have been reduced over time. Secondly, traces of gene flow (by long-distance dispersal) among neighboring regions, that is, among Dutch and German populations, might have counteracted the loss of genetic diversity due to genetic drift. From ringing data, there is further evidence of a steady exchange of breeding birds among neighboring Dutch populations (Both et al. 2006) and an influx of long-distance immigrants from the British Isles into Dutch breeding populations (Both et al. 2012). Thirdly, nest-site fidelity with respect to the place of birth was shown to be less pronounced in marginal Western European pied flycatcher populations than elsewhere in Europe (Winkel 1982). Thus, it is probable that Dutch pied flycatchers were able to compensate at least partly the loss of genetic diversity via gene flow with Western and Central Europe populations.

### Variation at the *Clock* gene locus

Variation in the number of poly-Q repeats at the *Clock* gene locus has been reported for several bird species on both levels, either among populations or within populations. This length variation seems to be a viable trait to test for an association between genetic and life-history trait variation, particularly with regard to changes in breeding phenology caused by recent climate change. The extremely low variation at the *Clock* gene locus in pied flycatchers is striking although the number of alleles identified (five in total, three up to five per population) is similar to that observed in other bird species (Johnsen et al. 2007; Liedvogel et al. 2009; Liedvogel and Sheldon 2010; Dor et al. 2011; Caprioli et al. 2012; Dor et al. 2012). Likewise, the numbers of 10 to 14 *Clock* poly-Q repeats are comparable to the numbers of tandem repeats in blue tits (*Cyanistes caeruleus*), great tits (*Parus major*), and bluethroats (*Luscinia svecica*) (Johnsen et al. 2007; Liedvogel et al. 2009; Liedvogel and Sheldon 2010), but exceed those found in the swallows of the genus *Tachycineta* (5 to 9) and the barn swallow (*Hirundo rustica*) (6 to 8) (Dor et al. 2011, 2012; Caprioli et al. 2012). The mean observed heterozygosity (*H*_o_ = 0.482) of pied flycatchers is in the same order of magnitude as values measured in blue tits (H_o_ = 0.489) although slightly higher than in bluethroats (*H*_o_ = 0.213) and differs considerably from the low values reported for great tits (*H*_o_ = 0.077) and barn swallows (*H*_o_ = 0.030).

Evidence for an association of *Clock* gene variation with both breeding latitude and breeding phenology of birds is scarce and inconsistent so far. A latitudinal cline of avian *Clock* ploy-Q variation was detected only in a nonmigratory species (blue tit; Johnsen et al. 2007), but there is counterevidence of such a cline in a number of other passerine species (Johnsen et al. 2007; Dor et al. 2011, 2012). Breeding latitudes of the pied flycatcher populations included in our study ranged from 41°5′ (Spain) to 69°24′ (Norway), and we could not confirm any correlation between latitude and *Clock* poly-Q length either. However, in North American dark-eyed juncos (*Junco hyemalis*), *Clock* poly-Q variation is significantly correlated with migration distance, and in the same study, species as well as in the blackcap (*Sylvia atricapilla*), the *ADCYAP1* locus, another gene of the ‘migratory gene package’ (Liedvogel et al. 2011), is similarly associated with both migratory distance and migratory restlessness (Müller et al. 2013; Peterson et al. 2013).

With respect to breeding phenology, intrapopulational analysis of the Spanish pied flycatchers yielded no significant correlation among *Clock* gene variation and laying dates, which is in concordance with results found in a great tit population in the UK (Liedvogel and Sheldon 2010) and in *Tachycineta* swallows (Dor et al. 2012). That contrasts the findings by Liedvogel et al. (2009) who documented a slight and not significant trend for earlier laying dates for blue tit females with fewer Q repeats (but see more recent analysis in Liedvogel et al. 2012). Similar results were reported for an Italian migrating barn swallow population (Caprioli et al. 2012). Apart from the timing of reproduction, there is evidence of an association of *Clock* gene variation with fitness-related traits such as clutch size and thus breeding success (Dor et al. 2012). Because of such conflicting results from independent studies of different avian model species, it seems that at this time no general conclusion can be drawn concerning an association between *Clock* genotypes and migratory behavior on the one hand and breeding phenology on the other hand.

### Stabilizing selection at the *Clock* gene locus

Among blue tit and bluethroat populations, significant associations between pairwise *F*_st_-values based on *Clock* gene allele frequency and those based on microsatellites were found (Johnsen et al. 2007). A different pattern was found in pied flycatcher (this study) and barn swallow (Dor et al. 2011) populations, that is, no association was detected and much lower differentiation at the *Clock* poly-Q locus than at microsatellite loci was found. In contrast to our expectation that genetic differentiation at candidate genes might be larger than at neutral loci, we observed the opposite pattern. The *Clock* gene in pied flycatchers seems to show no sign of neutral evolution, but a very low differentiation among populations compared with neutral markers (Fig. 5B). Similarly, intraspecific variation in two further candidate genes coding for a potentially sexually selected trait in the pied flycatcher (UV plumage coloration and visual perception of that trait) was recently demonstrated to deviate from the expectation of neutral evolution, too (Lehtonen et al. 2012). As a consequence, these genes and the avian *Clock* gene locus might well underlie stabilizing selection in the pied flycatcher. Equivalent to comparisons of neutral markers with phenotypic (*P*_ST_) or quantitative (*Q*_ST_) differentiation, lower differentiation in potentially selected traits or genes than in neutral loci suggests that natural selection must be favoring the same mean phenotype in different populations (Merilä and Cnorkrak 2001; Goudet and Martin 2007). Analyses of genetic differentiation among barn swallow populations revealed patterns of stabilizing selection as found in pied flycatchers (Dor et al. 2011). The observed spatial and temporal variation in migration and breeding phenology in pied flycatchers (Both et al. 2004) cannot solely be explained by variation at the *Clock* gene locus.

So far, it is not clear whether adaptation of migration and breeding time to rapid environmental changes is restricted through limited phenotypic plasticity and/or a lack of genetic variation on which selection can act. The increased selection for breeding date as observed in several long-term population studies (Both and Visser 2001; Goodenough et al. 2011) may not result in an adaptive response if the variation in breeding date has no genetic background. Adaptation may work through immigration of individuals of more southern origin that disperse northwards and thereby introduce genes for earlier timing (Edelaar et al. 2008). Indeed, in pied flycatchers, more southern populations migrate earlier in spring (Both 2010), but whether this earlier timing is genetically coded or the result of ontogeny is still unclear. Our results suggest that the *Clock* gene variation considered here is not a good candidate for such an adaptive process because neither between- nor within-population variation was related to variation in timing. The general lack of geographical variation in neutral genetic markers suggests that dispersal is an important process in this and other species of long-distance migrants, which would swamp most of the declines in genetic diversity in shrinking populations. This observation poses the question how much of the observed local phenotypic variation has a genetic origin. In fact, in the Spanish pied flycatcher population included in our analysis, spring arrival of males and breeding dates are shaped by a mixed-mating strategy of this species (sexual selection was suggested to be the main driving force of protandry; Canal et al. 2012). Identification of more candidate genes may help to unravel these processes, as will experiments in which individuals are translocated to breed in distant places and which consider how their offspring will behave relative to the local population (Burger and Both 2011).

## Data Accessibility

DNA sequences have been deposited at GenBank under accession numbers KF616865 – KF617193 for the mitochondrial control region and under numbers KF617194 – KF617224 for the *Clock* poly-Q alleles; sequence alignments, microsatellite data and *Clock* gene data available from the Dryad Digital Repository: http://doi.org/10.5061/dryad.6dc6k.

## Supporting Information

**Table S1.** Overview of samples used in this study with information regarding sample ID, source of DNA, specimen ID of the museum samples, geographic origin, sampling date, sex and inclusion or exclusion in population genetic analyses.

**Table S2.** Microsatellite loci (according to Leder et al. 2008), forward and reverse primer sequences (5 - 3'), labeling dye and final primer concentrations used for the multiplex PCR.

**Table S3.** Pairwise *F*_st_ comparisons between all populations: A) based on control region, B) based on microsatellite loci and C) based on clock gene loci.

**Table S4.** Mean number of *Clock*-gene Q repeats in male and female pied flycatchers (*F. hypoleuca*) found in different populations over space and time (both alleles added for each individual); n.a. = not analyzed separately for sexes due to a large number of nestling specimens without sex determination.

**Figure S1.** Median joining mtDNA haplotype network of recent European *F. hypoleuca* populations.

**Figure S2.** Barplot of a Bayesian clustering analysis using Structure, of recent *F. hypoleuca* individuals for the most likely K (4).
