# Supplementary material for: Differentiation in neutral genes and a candidate gene in the pied flycatcher: using biological archives to track global climate change
Source: Ecol Evol. 2013 Nov 1;3(14):4799–814. doi: 10.1002/ece3.855 (PMC3867912; doi:10.1002/ece3.855)
Supplement: Supplementary file 1 [file ece30003-4799-SD1.doc]

**Table S1**. Overview of samples used in this study with information regarding sample ID, source of DNA, specimen ID of the museum samples, geographic origin, sampling date, sex and inclusion or exclusion in population genetic analyses. Museum acronyms: MTD_C = Senckenberg Natural History Collections Dresden (SNSD; subcollections: MTD_Bährm= coll. Bährmann; MTD_Schlegel= coll. feathers Schlegel; MAR= tissue collection J. Martens), SNMG = Senckenberg Natural History Collections Görlitz, RMNH.AVES = Naturalis Biodiversity Center, NRM = Swedish Museum of Natural History Stockholm, ZMA.AVES = Zoological Museum Amsterdam, BMNH= British Museum of Natural History, Bird Collection Tring; material: DNA extracts of original material from Lethonen et al. (2009) were obtained from either blood or feather sample; * = feather samples housed at SNSD. Coordinates of sampling sites given in degrees and minutes (latitude: N°N´, longitude: E°,E´); juv. = juvenile; pull. = pullus; CR = control region (n= 343); MS = microsatellite (n= 278); CG = *Clock* gene (n= 279); + = included in analysis; - = not included in analysis (either PCR or sequencing failed, or less than 8 microsatellites amplified).

| **sample** | **material** | | | **specimen no** | **country** | **locality** | **N°** | **N´** | **E°** | **E´** | **date** | **sex** | **CR** | **MS** | **CG** |
| --- | --- | --- | --- | --- | --- | --- | --- | --- | --- | --- | --- | --- | --- | --- | --- |
| 1 | DNA extract | | | - | Norway | Skibotn | 69 | 24 | 20 | 16 | 2007 | female | + | + | + |
| 3 | DNA extract | | | - | Norway | Skibotn | 69 | 24 | 20 | 16 | 2007 | female | + | + | + |
| 5 | DNA extract | | | - | Norway | Skibotn | 69 | 24 | 20 | 16 | 2007 | female | + | + | + |
| 7 | DNA extract | | | - | Norway | Skibotn | 69 | 24 | 20 | 16 | 2007 | female | + | + | + |
| 8 | DNA extract | | | - | Norway | Skibotn | 69 | 24 | 20 | 16 | 2007 | female | + | + | + |
| 11 | DNA extract | | | - | Norway | Skibotn | 69 | 24 | 20 | 16 | 2007 | male | + | + | + |
| 12 | DNA extract | | | - | Norway | Skibotn | 69 | 24 | 20 | 16 | 2007 | male | + | + | + |
| 13 | DNA extract | | | - | Norway | Skibotn | 69 | 24 | 20 | 16 | 2007 | male | + | + | + |
| 15 | DNA extract | | | - | Norway | Skibotn | 69 | 24 | 20 | 16 | 2007 | male | + | + | + |
| 17 | DNA extract | | | - | Norway | Skibotn | 69 | 24 | 20 | 16 | 2007 | male | + | + | + |
| 25 | DNA extract | | | - | Norway | Skibotn | 69 | 24 | 20 | 16 | 2007 | male | + | + | + |
| 31 | DNA extract | | | - | Norway | Skibotn | 69 | 24 | 20 | 16 | 2007 | male | + | + | + |
| 35 | DNA extract | | | - | Norway | Skibotn | 69 | 24 | 20 | 16 | 2007 | male | + | + | + |
| 40 | DNA extract | | | - | Norway | Skibotn | 69 | 24 | 20 | 16 | 2007 | male | + | + | + |
| 42 | DNA extract | | | - | Norway | Skibotn | 69 | 24 | 20 | 16 | 2007 | male | + | + | + |
| 45 | DNA extract | | | - | Norway | Skibotn | 69 | 24 | 20 | 16 | 2007 | male | + | + | + |
| HL9943 | DNA extract | | | - | Finland | Ruissalo | 60 | 26 | 22 | 6 | 2005-2006 | female | + | + | + |
| HL79349 | DNA extract | | | - | Finland | Ruissalo | 60 | 26 | 22 | 6 | 2005-2006 | male | + | + | + |
| HL79352 | DNA extract | | | - | Finland | Ruissalo | 60 | 26 | 22 | 6 | 2005-2006 | female | + | + | + |
| JL763343 | DNA extract | | | - | Finland | Ruissalo | 60 | 26 | 22 | 6 | 2005-2006 | male | + | + | + |
| LL15255 | DNA extract | | | - | Finland | Ruissalo | 60 | 26 | 22 | 6 | 2005-2006 | male | + | + | + |
| LL15292 | DNA extract | | | - | Finland | Ruissalo | 60 | 26 | 22 | 6 | 2005-2006 | female | + | + | + |
| LL15293 | DNA extract | | | - | Finland | Ruissalo | 60 | 26 | 22 | 6 | 2005-2006 | female | + | + | + |
| LL15364 | DNA extract | | | - | Finland | Ruissalo | 60 | 26 | 22 | 6 | 2005-2006 | male | + | + | + |
| LL15294 | DNA extract | | | - | Finland | Ruissalo | 60 | 26 | 22 | 6 | 2005-2006 | female | + | + | + |
| LL15372 | DNA extract | | | - | Finland | Ruissalo | 60 | 26 | 22 | 6 | 2005-2006 | male | + | + | + |
| LL15417 | DNA extract | | | - | Finland | Ruissalo | 60 | 26 | 22 | 6 | 2005-2006 | male | + | + | + |
| LL15419 | DNA extract | | | - | Finland | Ruissalo | 60 | 26 | 22 | 6 | 2005-2006 | male | + | + | + |
| TL8749 | DNA extract | | | - | Finland | Ruissalo | 60 | 26 | 22 | 6 | 2005-2006 | female | + | + | + |
| TL8763 | DNA extract | | | - | Finland | Ruissalo | 60 | 26 | 22 | 6 | 2005-2006 | female | + | + | + |
| TL8783 | DNA extract | | | - | Finland | Ruissalo | 60 | 26 | 22 | 6 | 2005-2006 | male | + | + | + |
| TL8784 | DNA extract | | | - | Finland | Ruissalo | 60 | 26 | 22 | 6 | 2005-2006 | male | + | + | + |
| 624Sev | blood | | | - | Spain | La Hiruela | 41 | 5 | 3 | 27 | 18.06.2006 | male | + | + | + |
| 647Sev | blood | | | - | Spain | La Hiruela | 41 | 5 | 3 | 27 | 03.06.2006 | female | + | + | + |
| 652Sev | blood | | | - | Spain | La Hiruela | 41 | 5 | 3 | 27 | 06.06.2006 | female | + | + | + |
| 656Sev | blood | | | - | Spain | La Hiruela | 41 | 5 | 3 | 27 | 07.06.2006 | female | + | + | + |
| 661Sev | blood | | | - | Spain | La Hiruela | 41 | 5 | 3 | 27 | 04.06.2006 | female | + | + | + |
| 672Sev | blood | | | - | Spain | La Hiruela | 41 | 5 | 3 | 27 | 11.06.2006 | female | + | + | + |
| 679Sev | blood | | | - | Spain | La Hiruela | 41 | 5 | 3 | 27 | 06.06.2006 | female | + | + | + |
| 688Sev | blood | | | - | Spain | La Hiruela | 41 | 5 | 3 | 27 | 13.06.2006 | female | + | + | + |
| 692Sev | blood | | | - | Spain | La Hiruela | 41 | 5 | 3 | 27 | 04.06.2006 | female | + | + | + |
| 693Sev | blood | | | - | Spain | La Hiruela | 41 | 5 | 3 | 27 | 04.06.2006 | female | + | + | + |
| 694Sev | blood | | | - | Spain | La Hiruela | 41 | 5 | 3 | 27 | 31.05.2006 | female | + | + | + |
| 695Sev | blood | | | - | Spain | La Hiruela | 41 | 5 | 3 | 27 | 26.06.2006 | female | + | + | + |
| 700Sev | blood | | | - | Spain | La Hiruela | 41 | 5 | 3 | 27 | 10.06.2006 | female | + | + | + |
| 703Sev | blood | | | - | Spain | La Hiruela | 41 | 5 | 3 | 27 | 07.06.2006 | female | + | + | + |
| 706Sev | blood | | | - | Spain | La Hiruela | 41 | 5 | 3 | 27 | 16.06.2006 | female | + | + | + |
| 712Sev | blood | | | - | Spain | La Hiruela | 41 | 5 | 3 | 27 | 04.06.2006 | female | + | + | + |
| 718Sev | blood | | | - | Spain | La Hiruela | 41 | 5 | 3 | 27 | 12.06.2006 | female | + | + | + |
| 724Sev | blood | | | - | Spain | La Hiruela | 41 | 5 | 3 | 27 | 26.06.2006 | female | + | + | + |
| 729Sev | blood | | | - | Spain | La Hiruela | 41 | 5 | 3 | 27 | 27.05.2006 | female | + | + | + |
| 731Sev | blood | | | - | Spain | La Hiruela | 41 | 5 | 3 | 27 | 26.06.2006 | female | + | + | + |
| 734Sev | blood | | | - | Spain | La Hiruela | 41 | 5 | 3 | 27 | 04.06.2006 | female | + | + | + |
| 736Sev | blood | | | - | Spain | La Hiruela | 41 | 5 | 3 | 27 | 05.06.2006 | female | + | + | + |
| 737Sev | blood | | | - | Spain | La Hiruela | 41 | 5 | 3 | 27 | 05.06.2006 | female | + | + | + |
| 738Sev | blood | | | - | Spain | La Hiruela | 41 | 5 | 3 | 27 | 05.06.2006 | female | + | + | + |
| 747Sev | blood | | | - | Spain | La Hiruela | 41 | 5 | 3 | 27 | 08.06.2006 | female | + | + | + |
| 770Sev | blood | | | - | Spain | La Hiruela | 41 | 5 | 3 | 27 | 14.06.2006 | female | + | + | + |
| 771Sev | blood | | | - | Spain | La Hiruela | 41 | 5 | 3 | 27 | 06.06.2006 | female | + | + | + |
| 774Sev | blood | | | - | Spain | La Hiruela | 41 | 5 | 3 | 27 | 12.06.2006 | male | + | + | + |
| 777Sev | blood | | | - | Spain | La Hiruela | 41 | 5 | 3 | 27 | 05.06.2006 | male | + | + | + |
| 778Sev | blood | | | - | Spain | La Hiruela | 41 | 5 | 3 | 27 | 01.06.2006 | male | + | + | + |
| 783Sev | blood | | | - | Spain | La Hiruela | 41 | 5 | 3 | 27 | 06.06.2006 | male | + | + | + |
| 788Sev | blood | | | - | Spain | La Hiruela | 41 | 5 | 3 | 27 | 03.06.2006 | male | + | + | + |
| 789Sev | blood | | | - | Spain | La Hiruela | 41 | 5 | 3 | 27 | 14.06.2006 | male | + | + | - |
| 797Sev | blood | | | - | Spain | La Hiruela | 41 | 5 | 3 | 27 | 14.06.2006 | male | + | + | + |
| 800Sev | blood | | | - | Spain | La Hiruela | 41 | 5 | 3 | 27 | 08.06.2006 | male | + | + | + |
| 804Sev | blood | | | - | Spain | La Hiruela | 41 | 5 | 3 | 27 | 04.06.2006 | male | + | + | + |
| 806Sev | blood | | | - | Spain | La Hiruela | 41 | 5 | 3 | 27 | 05.06.2006 | male | + | + | + |
| 809Sev | blood | | | - | Spain | La Hiruela | 41 | 5 | 3 | 27 | 11.06.2005 | male | + | + | + |
| 818Sev | blood | | | - | Spain | La Hiruela | 41 | 5 | 3 | 27 | 08.06.2006 | male | + | + | - |
| 820Sev | blood | | | - | Spain | La Hiruela | 41 | 5 | 3 | 27 | 08.06.2006 | male | + | + | + |
| 844Sev | blood | | | - | Spain | La Hiruela | 41 | 5 | 3 | 27 | 14.06.2006 | male | + | + | + |
| 845Sev | blood | | | - | Spain | La Hiruela | 41 | 5 | 3 | 27 | 15.06.2006 | male | + | + | + |
| 855Sev | blood | | | - | Spain | La Hiruela | 41 | 5 | 3 | 27 | 04.06.2006 | male | + | + | + |
| 859Sev | blood | | | - | Spain | La Hiruela | 41 | 5 | 3 | 27 | 05.06.2006 | male | + | + | + |
| 861Sev | blood | | | - | Spain | La Hiruela | 41 | 5 | 3 | 27 | 06.06.2006 | male | + | + | + |
| C18984 | toe pad | | | MTD_C18984 | Germany | Dresdner Heide | 51 | 54 | 13 | 48 | 12.05.1904 | male juv.. | + | - | + |
| C18985 | toe pad | | | MTD_C18985 | Germany | Dresdner Heide | 51 | 54 | 13 | 48 | 12.05.1904 | female | + | - | - |
| C37570 | toe pad | | | MTD_C37570 | Germany | Schöneiche, Brandenburg | 52 | 13 | 13 | 31 | 02.05.1901 | male | + | - | + |
| C40303 | toe pad | | | MTD_C40303 | Germany | Pillnitz near Dresden | 51 | 1 | 13 | 53 | 29.05.1901 | female | + | - | + |
| C37571 | toe pad | | | MTD_C37571 | Germany | Schöneiche, Brandenburg | 52 | 13 | 13 | 31 | 03.05.1901 | female | + | - | - |
| C23011 | toe pad | | | MTD_C23011 | Germany | Wahnsdorf near Dresden | 51 | 7 | 13 | 40 | 16.05.1923 | male juv.. | + | - | - |
| C40932 | toe pad | | | MTD_C40932 | Germany | Eythra near Leipzig | 51 | 14 | 12 | 18 | 10.05.1919 | male | + | - | - |
| C32299 | toe pad | | | MTD_C32299 | Germany | Eythra near Leipzig | 51 | 14 | 12 | 18 | 21.05.1920 | male juv.. | + | - | + |
| C32292 | toe pad | | | MTD_C32292 | Germany | Leipzig-Connewitz | 51 | 17 | 12 | 19 | 09.05.1920 | female | + | - | + |
| C32300 | toe pad | | | MTD_C32300 | Germany | Eythra near Leipzig | 51 | 14 | 12 | 18 | 06.05.1923 | male juv.. | + | - | + |
| C32294 | toe pad | | | MTD_C32294 | Germany | Leipzig-Schönefeld | 51 | 22 | 12 | 25 | 08.05.1925 | male | + | - | + |
| C40595 | toe pad | | | MTD_C40595 | Germany | Kriebstein, Sachsen | 51 | 3 | 13 | 1 | 26.05.1935 | male | + | - | - |
| Bährm2977 | toe pad | | | MTD_Bährm2977 | Germany | Lauchhammer | 51 | 30 | 13 | 48 | 04.05.1941 | female | + | + | + |
| Bährm2986 | toe pad | | | MTD_Bährm2986 | Germany | Grünewalde | 51 | 58 | 11 | 55 | 04.05.1947 | male | + | + | + |
| Bährm2987 | toe pad | | | MTD_Bährm2987 | Germany | Grünewalde | 51 | 58 | 11 | 55 | 04.05.1947 | male | + | + | + |
| C41605 | toe pad | | | MTD_C41605 | Germany | Tharandt | 50 | 58 | 13 | 34 | 22.06.1947 | male | + | - | - |
| Bährm2988 | toe pad | | | MTD_Bährm2988 | Germany | Grünewalde | 51 | 58 | 11 | 55 | 03.05.1947 | male | + | + | + |
| C46150 | toe pad | | | MTD_C46150 | Germany | Pillnitz near Dresden | 51 | 1 | 13 | 53 | June 1947 | female | + | + | - |
| Bährm2989 | toe pad | | | MTD_Bährm2989 | Germany | Grünewalde | 51 | 58 | 11 | 55 | 03.05.1947 | male | + | + | + |
| Bährm2990 | toe pad | | | MTD_Bährm2990 | Germany | Lauchhammer | 51 | 30 | 13 | 48 | 02.05.1948 | male | + | + | + |
| C40902 | toe pad | | | MTD_C40902 | Germany | Tharandt | 50 | 58 | 13 | 34 | 20.04.1948 | male | + | - | + |
| C41607 | toe pad | | | MTD_C41607 | Germany | Tharandt | 50 | 58 | 13 | 34 | 03.06.1948 | female | + | + | + |
| C41608 | toe pad | | | MTD_C41608 | Germany | Tharandt | 50 | 58 | 13 | 34 | 20.06.1948 | female | + | - | + |
| C40904 | toe pad | | | MTD_C40904 | Germany | Tharandt | 50 | 58 | 13 | 34 | 26.04.1948 | male | + | - | - |
| C40903 | toe pad | | | MTD_C40903 | Germany | Tharandt | 50 | 58 | 13 | 34 | 28.06.1948 | male | + | - | - |
| C41604 | toe pad | | | MTD_C41604 | Germany | Tharandt | 50 | 58 | 13 | 34 | 22.04.1949 | male | + | + | + |
| C40905 | toe pad | | | MTD_C40905 | Germany | Tharandt | 50 | 58 | 13 | 34 | 22.04.1949 | female | + | + | + |
| C46145 | toe pad | | | MTD_C46145 | Germany | Pillnitz near Dresden | 51 | 1 | 13 | 53 | 08.05.1949 | female | + | + | + |
| C46124 | toe pad | | | MTD_C46124 | Germany | Dresden-Hosterwitz | 51 | 1 | 13 | 51 | 07.06.1951 | male | + | + | + |
| C46130 | toe pad | | | MTD_C46130 | Germany | Dresden-Hosterwitz | 51 | 1 | 13 | 51 | 15.05.1951 | male | + | + | + |
| C46141 | toe pad | | | MTD_C46141 | Germany | Dresden-Hosterwitz | 51 | 1 | 13 | 51 | 11.06.1951 | - | + | + | + |
| C49284 | toe pad | | | MTD_C49284 | Germany | Dresden-Hellerau | 51 | 7 | 13 | 45 | June 1951 | female? | + | + | + |
| C46140 | toe pad | | | MTD_C46140 | Germany | Dresden-Hosterwitz | 51 | 1 | 13 | 51 | 11.06.1951 | ? | + | + | - |
| C46148 | toe pad | | | MTD_C46148 | Germany | Pillnitz near Dresden | 51 | 1 | 13 | 53 | June 1951 | male | + | + | + |
| C46154 | toe pad | | | MTD_C46154 | Germany | Graupa | 51 | 0 | 13 | 55 | 04.06.1951 | male | + | + | + |
| C46151 | toe pad | | | MTD_C46151 | Germany | Pillnitz near Dresden | 51 | 1 | 13 | 53 | 04.06.1952 | female | + | + | - |
| C46153 | toe pad | | | MTD_C46153 | Germany | Graupa | 51 | 0 | 13 | 55 | 27.05.1952 | male | + | - | + |
| C46152 | toe pad | | | MTD_C46152 | Germany | Graupa | 51 | 0 | 13 | 55 | 09.06.1954 | female | + | + | + |
| C49283 | toe pad | | | MTD_C49283 | Germany | Lommatzsch | 51 | 12 | 13 | 18 | 10.05.1967 | pull.. | + | + | + |
| C64612 | toe pad | | | MTD_C64612 | Germany | Lomnitz near Dresden | 51 | 11 | 13 | 54 | 17.05.1967 | - | + | + | - |
| C49282 | toe pad | | | MTD_C49282 | Germany | Jena | 50 | 55 | 11 | 35 | 04.05.1958 | female | - | + | + |
| C48422 | toe pad | | | MTD_C48422 | Germany | Moritzburg near Dresden | 51 | 10 | 13 | 40 | 18.06.1969 | juv. | + | + | + |
| C49285 | toe pad | | | MTD_C49285 | Germany | Radebeul | 51 | 6 | 13 | 40 | 26.07.1970 | male? | + | + | + |
| S1670 | feather, dry | | | MTD_Schlegel1670/1 | Germany | Annaberg, Vogtland | 50 | 34 | 13 | 0 | 13.05.1970 | - | + | - | - |
| S1671 | feather, dry | | | MTD_Schlegel1671/1 | Germany | Annaberg, Vogtland | 50 | 34 | 13 | 0 | 14.06.1970 | - | + | - | - |
| S1672 | toe pad | | | MTD_Schlegel1672/1 | Germany | Annaberg, Vogtland | 50 | 34 | 13 | 0 | 16.05.1971 | - | + | - | - |
| S1673 | feather, dry | | | MTD_Schlegel1673/1 | Germany | Scharfenstein, Erzgebirge | 50 | 42 | 13 | 3 | 30.06.1971 | - | + | - | - |
| C49281 | toe pad | | | MTD_C49281 | Germany | Moritzburg near Dresden | 51 | 10 | 13 | 40 | 30.04.1977 | male | + | + | + |
| C49280 | toe pad | | | MTD_C49280 | Germany | Moritzburg near Dresden | 51 | 10 | 13 | 40 | 30.04.1971 | female | + | + | + |
| Bährm2978 | toe pad | | | MTD_Bährm2978 | Germany | Schraden | 51 | 21 | 13 | 41 | 10.05.1972 | female | + | + | - |
| 762SA72 | toe pad | | | MTD_Schlegel1359/2 | Germany | Annaberg, Humpeltal | 50 | 34 | 13 | 0 | 28.06.1972 | - | + | + | - |
| C63611 | toe pad | | | MTD_C63611 | Germany | Borsberg near Dresden | 51 | 1 | 13 | 54 | 17.05.1973 | male | + | + | - |
| C49287 | toe pad | | | MTD_C49287 | Germany | Moritzburg near Dresden | 51 | 10 | 13 | 40 | 24.07.1973 | male? | + | + | + |
| C45969 | toe pad | | | MTD_C45969 | Germany | Graupa near Dresden | 51 | 0 | 13 | 55 | June 1974 | male | + | + | + |
| C53112 | toe pad | | | MTD_C53112 | Germany | Rossendorf near Dresden | 51 | 3 | 13 | 56 | 18.05.1995 | male | + | + | - |
| MAR3743 | toe pad | | | MTD_C61607 | Germany | Liegau-Augustusbad | 51 | 8 | 13 | 53 | 03.07.2000 | juv. | + | + | + |
| SC299 | feather, dry | | | MTD_Schlegel299/4 | Germany | Wiesa, Erzgebirge | 50 | 37 | 13 | 1 | 06.05.2003 | - | + | - | + |
| 761SA04 | feather, dry | | | MTD_Schlegel583/4 | Germany | Annaberg, Humpeltal | 50 | 34 | 13 | 0 | 15.05.2004 | - | + | + | - |
| 5271SA05 | tissue | | | SNMG_A.1310 | Germany | Gaußig, Gut Sommereichen | 51 | 8 | 14 | 19 | 19.07.2005 | male | + | + | - |
| 763SA05 | toe pad | | | MTD_Schlegel737/4 | Germany | Annaberg, Vogtland | 50 | 34 | 13 | 0 | 03.07.2005 | - | + | + | - |
| 764SA04 | toe pad | | | MTD_Schlegel582/4 | Germany | Annaberg, Humpeltal | 50 | 34 | 13 | 0 | 04.05.2004 | - | + | + | - |
| MAR6695 | tissue | | | SNMG_A.1633 | Germany | Kreba | 51 | 21 | 14 | 41 | 03.06.2007 | - | + | - | + |
| MAR6696 | tissue | | | SNMG_A.1634 | Germany | Kreba | 51 | 21 | 14 | 41 | 03.06.2007 | - | + | + | + |
| MAR6697 | tissue | | | SNMG_A.1635 | Germany | Kreba | 51 | 21 | 14 | 41 | 03.06.2007 | - | + | + | + |
| MAR6698 | tissue | | | SNMG_A.1636 | Germany | Kreba | 51 | 21 | 14 | 41 | 03.06.2007 | - | + | + | + |
| MAR8263 | tissue | | | SNMG_A.1988 | Germany | Deschka | 51 | 16 | 15 | 2 | 04.05.2010 | male | + | + | + |
| MTD728 | tissue | | | - | Germany | Steckby, Sachsen-Anhalt | 51 | 53 | 12 | 1 | 2008 | pull. | + | + | + |
| MTD730 | tissue | | | - | Germany | Steckby, Sachsen-Anhalt | 51 | 53 | 12 | 1 | 2008 | pull. | + | + | + |
| MTD731 | tissue | | | - | Germany | Steckby, Sachsen-Anhalt | 51 | 53 | 12 | 1 | 2008 | pull. | + | + | + |
| MTD732 | tissue | | | - | Germany | Steckby, Sachsen-Anhalt | 51 | 53 | 12 | 1 | 2008 | pull. | + | + | + |
| MTD735 | feather, alcohol | | | - | Germany | Steckby, Sachsen-Anhalt | 51 | 53 | 12 | 1 | 2008 | pull. | + | + | + |
| MTD736 | feather, alcohol | | | - | Germany | Steckby, Sachsen-Anhalt | 51 | 53 | 12 | 1 | 2008 | pull. | + | + | + |
| MTD737 | feather, alcohol | | | - | Germany | Steckby, Sachsen-Anhalt | 51 | 53 | 12 | 1 | 2008 | female | + | + | + |
| MTD738 | feather, alcohol | | | - | Germany | Steckby, Sachsen-Anhalt | 51 | 53 | 12 | 1 | 2008 | female | + | + | + |
| MTD739 | feather, alcohol | | | - | Germany | Steckby, Sachsen-Anhalt | 51 | 53 | 12 | 1 | 2008 | pull. | + | + | + |
| MTD740 | feather, alcohol | | | - | Germany | Steckby, Sachsen-Anhalt | 51 | 53 | 12 | 1 | 2008 | pull. | + | + | + |
| MTD741 | feather, alcohol | | | - | Germany | Steckby, Sachsen-Anhalt | 51 | 53 | 12 | 1 | 2008 | pull. | + | + | + |
| MTD742 | feather, alcohol | | | - | Germany | Steckby, Sachsen-Anhalt | 51 | 53 | 12 | 1 | 2008 | pull. | + | + | + |
| MTD743 | feather, alcohol | | | - | Germany | Steckby, Sachsen-Anhalt | 51 | 53 | 12 | 1 | 2008 | pull. | + | + | + |
| MTD745 | feather, alcohol | | | - | Germany | Steckby, Sachsen-Anhalt | 51 | 53 | 12 | 1 | 2008 | pull. | + | + | + |
| MTD746 | feather, alcohol | | | - | Germany | Steckby, Sachsen-Anhalt | 51 | 53 | 12 | 1 | 2008 | pull. | + | + | + |
| MTD744 | feather, alcohol | | | - | Germany | Steckby, Sachsen-Anhalt | 51 | 53 | 12 | 1 | 2008 | pull. | + | + | + |
| MTD747 | feather, alcohol | | | - | Germany | Steckby, Sachsen-Anhalt | 51 | 53 | 12 | 1 | 2008 | pull. | + | + | + |
| MTD748 | feather, alcohol | | | - | Germany | Steckby, Sachsen-Anhalt | 51 | 53 | 12 | 1 | 2008 | pull. | + | + | + |
| MTD749 | feather, alcohol | | | - | Germany | Steckby, Sachsen-Anhalt | 51 | 53 | 12 | 1 | 2008 | pull. | + | + | + |
| MTD751 | feather, alcohol | | | - | Germany | Steckby, Sachsen-Anhalt | 51 | 53 | 12 | 1 | 2008 | pull. | + | + | + |
| MTD752 | feather, alcohol | | | - | Germany | Steckby, Sachsen-Anhalt | 51 | 53 | 12 | 1 | 2008 | pull. | + | + | + |
| MTD753 | feather, alcohol | | | - | Germany | Steckby, Sachsen-Anhalt | 51 | 53 | 12 | 1 | 2008 | pull. | + | + | + |
| MTD754 | feather, alcohol | | | - | Germany | Steckby, Sachsen-Anhalt | 51 | 53 | 12 | 1 | 2008 | pull. | + | + | + |
| MTD755 | feather, alcohol | | | - | Germany | Steckby, Sachsen-Anhalt | 51 | 53 | 12 | 1 | 2008 | pull. | + | + | + |
| MTD757 | feather, alcohol | | | - | Germany | Steckby, Sachsen-Anhalt | 51 | 53 | 12 | 1 | 2008 | pull. | + | + | + |
| MTD758 | feather, alcohol | | | - | Germany | Steckby, Sachsen-Anhalt | 51 | 53 | 12 | 1 | 2008 | pull. | + | + | + |
| AJ4502 | feather, dry | | | AJ4502ST* | The Netherlands | Staphorst | 52 | 39 | 6 | 12 | 03.06.2008 | female | + | + | + |
| AF37356 | feather, dry | | | AF37356ST* | The Netherlands | Staphorst | 52 | 39 | 6 | 12 | 03.06.2008 | male | + | + | + |
| AP57416 | feather, dry | | | AP57416ST* | The Netherlands | Staphorst | 52 | 39 | 6 | 12 | 03.06.2008 | female | + | + | + |
| AP57423 | feather, dry | | | AP57423ST* | The Netherlands | Staphorst | 52 | 39 | 6 | 12 | 03.06.2008 | female | + | + | + |
| AP57427 | feather, dry | | | AP57427ST* | The Netherlands | Staphorst | 52 | 39 | 6 | 12 | 03.06.2008 | female | + | + | + |
| AP57431 | feather, dry | | | AP57431ST* | The Netherlands | Staphorst | 52 | 39 | 6 | 12 | 05.06.2008 | female | + | + | + |
| AP57432 | feather, dry | | | AP57432ST* | The Netherlands | Staphorst | 52 | 39 | 6 | 12 | 05.06.2008 | female | + | + | + |
| AP57433 | feather, dry | | | AP57433ST* | The Netherlands | Staphorst | 52 | 39 | 6 | 12 | 05.06.2008 | female | + | + | - |
| AD77167 | feather, dry | | | AD77167ST* | The Netherlands | Staphorst | 52 | 39 | 6 | 12 | 03.06.2008 | female | + | + | + |
| AL87092 | feather, dry | | | AL87092ST* | The Netherlands | Staphorst | 52 | 39 | 6 | 12 | 03.06.2008 | female | + | + | + |
| AH74393 | feather, dry | | | AH74393ST* | The Netherlands | Staphorst | 52 | 39 | 6 | 12 | June 2008 | male | + | + | + |
| AH80516 | feather, dry | | | AH80516ST* | The Netherlands | Staphorst | 52 | 39 | 6 | 12 | June 2008 | male | + | + | + |
| AK94795 | feather, dry | | | AK94795ST* | The Netherlands | Staphorst | 52 | 39 | 6 | 12 | June 2008 | female | + | + | + |
| AL70607 | feather, dry | | | AL70607ST* | The Netherlands | Staphorst | 52 | 39 | 6 | 12 | June 2008 | male | + | + | + |
| AL71842 | feather, dry | | | AL71842ST* | The Netherlands | Staphorst | 52 | 39 | 6 | 12 | June 2008 | male | + | + | - |
| AL72669 | feather, dry | | | AL72669ST* | The Netherlands | Staphorst | 52 | 39 | 6 | 12 | June 2008 | female | + | + | + |
| AL75268 | feather, dry | | | AL75268ST* | The Netherlands | Staphorst | 52 | 39 | 6 | 12 | June 2008 | male | + | + | + |
| AL75334 | feather, dry | | | AL75334ST* | The Netherlands | Staphorst | 52 | 39 | 6 | 12 | June 2008 | female | + | + | + |
| AL75519 | feather, dry | | | AL75519ST* | The Netherlands | Staphorst | 52 | 39 | 6 | 12 | June 2008 | male | + | + | + |
| AL75914 | feather, dry | | | AL75914ST* | The Netherlands | Staphorst | 52 | 39 | 6 | 12 | June 2008 | female | + | + | + |
| AL75968 | feather, dry | | | AL75968ST* | The Netherlands | Staphorst | 52 | 39 | 6 | 12 | June 2008 | female | + | + | + |
| AN61159 | feather, dry | | | AN61159ST* | The Netherlands | Staphorst | 52 | 39 | 6 | 12 | June 2008 | male | + | + | + |
| AN61161 | feather, dry | | | AN61161ST* | The Netherlands | Staphorst | 52 | 39 | 6 | 12 | June 2008 | female | + | + | + |
| AN61162 | feather, dry | | | AN61162ST* | The Netherlands | Staphorst | 52 | 39 | 6 | 12 | June 2008 | female | + | + | + |
| AN61163 | feather, dry | | | AN61163ST* | The Netherlands | Staphorst | 52 | 39 | 6 | 12 | June 2008 | female | + | + | + |
| AN61165 | feather, dry | | | AN61165ST* | The Netherlands | Staphorst | 52 | 39 | 6 | 12 | June 2008 | male | + | + | + |
| AN65619 | feather, dry | | | AN65619ST* | The Netherlands | Staphorst | 52 | 39 | 6 | 12 | June 2008 | female | + | + | + |
| AN65952 | feather, dry | | | AN65952ST* | The Netherlands | Staphorst | 52 | 39 | 6 | 12 | June 2008 | female | + | + | + |
| AN68196 | feather, dry | | | AN68196ST* | The Netherlands | Staphorst | 52 | 39 | 6 | 12 | June 2008 | female | + | + | + |
| AP26152 | feather, dry | | | AP26152ST* | The Netherlands | Staphorst | 52 | 39 | 6 | 12 | June 2008 | female | + | + | + |
| Ak92070 | feather, dry | | | Ak92070ST* | The Netherlands | Staphorst | 52 | 39 | 6 | 12 | June 2008 | male | + | + | + |
| AD4875 | feather, dry | | | AD4875De* | The Netherlands | Deelerwoud | 52 | 5 | 5 | 55 | 08.05.2003 | female | + | + | + |
| AF22146 | feather, dry | | | AF22146De* | The Netherlands | Deelerwoud | 52 | 5 | 5 | 55 | May 2003 | female | + | + | + |
| AF22151 | feather, dry | | | AF22151De* | The Netherlands | Deelerwoud | 52 | 5 | 5 | 55 | 07.05.2003 | female | + | + | + |
| AE22158 | feather, dry | | | AE22158De* | The Netherlands | Deelerwoud | 52 | 5 | 5 | 55 | 09.05.2003 | female | + | + | + |
| AF22162 | feather, dry | | | AF22162De* | The Netherlands | Deelerwoud | 52 | 5 | 5 | 55 | 25.05.2003 | ? | + | + | + |
| AF22206 | feather, dry | | | AF22206De* | The Netherlands | Deelerwoud | 52 | 5 | 5 | 55 | 09.05.2003 | female | + | + | + |
| AF22220 | feather, dry | | | AF22220De* | The Netherlands | Deelerwoud | 52 | 5 | 5 | 55 | 29.05.2003 | male | + | + | + |
| AE23026 | feather, dry | | | AE23026De* | The Netherlands | Deelerwoud | 52 | 5 | 5 | 55 | May 2003 | female | + | + | + |
| AE23104 | feather, dry | | | AE23104De* | The Netherlands | Deelerwoud | 52 | 5 | 5 | 55 | 08.05.2003 | female | + | + | + |
| A626554 | feather, dry | | | A626554De* | The Netherlands | Deelerwoud | 52 | 5 | 5 | 55 | 25.06.2003 | male | + | + | + |
| AA88106 | feather, dry | | | AA88106De* | The Netherlands | Deelerwoud | 52 | 5 | 5 | 55 | May 2003 | female | + | + | + |
| AA98646 | feather, dry | | | AA98646De* | The Netherlands | Deelerwoud | 52 | 5 | 5 | 55 | 28.05.2003 | male | + | + | + |
| AB75719 | feather, dry | | | AB75719De* | The Netherlands | Deelerwoud | 52 | 5 | 5 | 55 | 25.06.2003 | female | + | + | + |
| AB75908 | feather, dry | | | AB75908De* | The Netherlands | Deelerwoud | 52 | 5 | 5 | 55 | 25.06.2003 | male | + | + | + |
| AD4289 | feather, dry | | | AD4289De* | The Netherlands | Deelerwoud | 52 | 5 | 5 | 55 | 08.05.2003 | female | + | + | + |
| AE3652 | feather, dry | | | AE3652De* | The Netherlands | Deelerwoud | 52 | 5 | 5 | 55 | 27.05.2003 | male | + | + | + |
| AF22052 | feather, dry | | | AF22052De* | The Netherlands | Deelerwoud | 52 | 5 | 5 | 55 | 29.05.2003 | male | + | + | + |
| AF22161 | feather, dry | | | AF22161De* | The Netherlands | Deelerwoud | 52 | 5 | 5 | 55 | May 2003 | male | + | + | + |
| AF22221 | feather, dry | | | AF22221De* | The Netherlands | Deelerwoud | 52 | 5 | 5 | 55 | 29.05.2003 | male | + | + | + |
| AF23118 | feather, dry | | | AF23118De* | The Netherlands | Deelerwoud | 52 | 5 | 5 | 55 | May 2003 | female | + | + | + |
| AG26532 | feather, dry | | | AG26532De* | The Netherlands | Deelerwoud | 52 | 5 | 5 | 55 | 03.05.2003 | male | + | + | + |
| F708262 | feather, dry | | | F708262De* | The Netherlands | Deelerwoud | 52 | 5 | 5 | 55 | May 2003 | male | + | + | + |
| F708433 | feather, dry | | | F708433De* | The Netherlands | Deelerwoud | 52 | 5 | 5 | 55 | 29.05.2003 | female | + | + | + |
| AP57417 | feather, dry | | | AP57417Ma* | The Netherlands | Markelo | 52 | 14 | 6 | 29 | 03.06.2008 | male | + | + | + |
| AP57418 | feather, dry | | | AP57418Ma* | The Netherlands | Markelo | 52 | 14 | 6 | 29 | 03.06.2008 | male | + | + | + |
| AP57419 | feather, dry | | | AP57419Ma* | The Netherlands | Markelo | 52 | 14 | 6 | 29 | 03.06.2008 | female | + | + | + |
| AP57421 | feather, dry | | | AP57421Ma* | The Netherlands | Markelo | 52 | 14 | 6 | 29 | 03.06.2008 | male | + | + | + |
| AP57426 | feather, dry | | | AP57426Ma* | The Netherlands | Markelo | 52 | 14 | 6 | 29 | 03.06.2008 | male | + | + | + |
| AL87049 | feather, dry | | | AL87049Ma* | The Netherlands | Markelo | 52 | 14 | 6 | 29 | 03.06.2008 | female | + | + | + |
| AL87373 | feather, dry | | | AL87373Ma* | The Netherlands | Markelo | 52 | 14 | 6 | 29 | 03.06.2008 | female | + | + | + |
| AL87052 | feather, dry | | | AL87052Ma* | The Netherlands | Markelo | 52 | 14 | 6 | 29 | 03.06.2008 | male | + | + | + |
| AH98430 | feather, dry | | | AH98430Ma* | The Netherlands | Markelo | 52 | 14 | 6 | 29 | 03.06.2008 | female | + | + | + |
| AL87539 | feather, dry | | | AL87539* | The Netherlands | Markelo | 52 | 14 | 6 | 29 | 03.06.2008 | male | - | + | + |
| NL9725 | toe pad | | | RMNH.AVES.9725 | The Netherlands | Rievendink, Brinkheurne, Winterswijk | 51 | 58 | 6 | 43 | 10.05.1941 | male | + | + | + |
| NL7170 | toe pad | | | ZMA.AVES.7170 | The Netherlands | Amelo, cemetery | 52 | 21 | 6 | 40 | 01.05.1943 | male | + | - | + |
| NL7171 | toe pad | | | ZMA.AVES.7171 | The Netherlands | Amelo, cemetery | 52 | 21 | 6 | 40 | 10.06.1943 | female | + | - | + |
| NL68314 | toe pad | | | RMNH.AVES.68314 | The Netherlands | Schalkhaar near Deventer | 52 | 16 | 6 | 12 | 13.06.1962 | male | + | + | + |
| NL45662 | toe pad | | | ZMA.AVES.45662 | The Netherlands | Bussum, North Holland | 52 | 16 | 5 | 12 | 04.05.1947 | male | + | - | + |
| NL7173 | toe pad | | | ZMA.AVES.7173 | The Netherlands | Zwolle, Meppelerweg "De Paddestoel" | 52 | 30 | 6 | 5 | 05.05.1947 | male | + | - | + |
| NL45661 | toe pad | | | ZMA.AVES.45661 | The Netherlands | Bussum, North Holland | 52 | 16 | 5 | 12 | 16.05.1944 | female | + | + | + |
| NL32119 | toe pad | | | RMNH.AVES.32119 | The Netherlands | Eefde | 52 | 10 | 6 | 13 | 01.07.1948 | pull. | + | + | + |
| NL58066 | toe pad | | | RMNH.AVES.58066 | The Netherlands | Kleine Noordijk, te WILP, Gelderland | 52 | 8 | 6 | 34 | 22.06.1949 | pull. | + | + | + |
| NL15793 | toe pad | | | RMNH.AVES.15793 | The Netherlands | Oud Schoonebeek | 52 | 39 | 6 | 53 | 04.05.1951 | male | + | + | + |
| NL32120 | toe pad | | | RMNH.AVES.32120 | The Netherlands | Eefde | 52 | 10 | 6 | 13 | 21.05.1952 | male | + | + | + |
| NL23896 | toe pad | | | RMNH.AVES.23896 | The Netherlands | Kleine Noordijk, Gelderland | 52 | 8 | 6 | 34 | 12.07.1955 | pull., female | + | + | + |
| NL32121 | toe pad | | | RMNH.AVES.32121 | The Netherlands | Warnsveld | 52 | 8 | 6 | 12 | 07.06.1956 | pull. | + | + | + |
| NL32122 | toe pad | | | RMNH.AVES.32122 | The Netherlands | Warnsveld | 52 | 8 | 6 | 12 | 07.06.1956 | pull. | + | - | + |
| NL32123 | toe pad | | | RMNH.AVES.32123 | The Netherlands | Warnsveld | 52 | 8 | 6 | 12 | 07.06.1956 | pull. | + | + | + |
| NL32124 | toe pad | | | RMNH.AVES.32124 | The Netherlands | Warnsveld | 52 | 8 | 6 | 12 | 07.06.1956 | pull. | + | + | + |
| NL14466 | toe pad | | | ZMA.AVES.14466 | The Netherlands | Amsterdam, Botanical Garden | 52 | 22 | 4 | 54 | 25.05.1959 | female | + | - | + |
| NL68313 | toe pad | | | RMNH.AVES.68313 | The Netherlands | Zwollerkerspel | 52 | 31 | 6 | 5 | 25.04.1960 | female | + | + | + |
| NL29934 | toe pad | | | RMNH.AVES.29934 | The Netherlands | Voorst | 52 | 14 | 6 | 5 | 09.05.1960 | female | + | + | + |
| NL29933 | toe pad | | | RMNH.AVES.29933 | The Netherlands | Voorst | 52 | 14 | 6 | 5 | 09.05.1960 | male | + | + | + |
| NL19007 | toe pad | | | ZMA.AVES.19007 | The Netherlands | Sieverdinck near Kotten, Winterswijk | 51 | 57 | 6 | 46 | 08.05.1966 | male | + | + | + |
| NL60205 | toe pad | | | RMNH.AVES.60205 | The Netherlands | Zeesse, Ommen | 52 | 31 | 6 | 25 | 24.05.1969 | female | + | + | + |
| NL60206 | toe pad | | | RMNH.AVES.60206 | The Netherlands | Zeesse, Ommen | 52 | 31 | 6 | 25 | 24.05.1969 | female | + | + | + |
| NL56189 | toe pad | | | ZMA.AVES.56189 | The Netherlands | Landgoed, Drakenburg, Hilversum | 52 | 14 | 5 | 11 | 07.05.1970 | male | + | + | + |
| NL68725 | toe pad | | | RMNH.AVES.68725 | The Netherlands | Ommen | 52 | 31 | 6 | 25 | 28.04.1972 | male | + | + | + |
| NL30859 | toe pad | | | ZMA.AVES.30859 | The Netherlands | Bennekom, Gelderland | 52 | 0 | 5 | 41 | end April 1973 | ? | + | - | - |
| NL30857 | toe pad | | | ZMA.AVES.30857 | The Netherlands | Keyenberg near Bennekom | 52 | 0 | 5 | 4 | 03.06.1973 | male | + | + | + |
| NL76882 | toe pad | | | RMNH.AVES.76882 | The Netherlands | Ommen | 52 | 31 | 6 | 25 | 15.05.1974 | male | + | + | + |
| NL76883 | toe pad | | | RMNH.AVES.76883 | The Netherlands | Ommen | 52 | 31 | 6 | 25 | 15.05.1974 | male | + | + | + |
| NL30853 | toe pad | | | ZMA.AVES.30853 | The Netherlands | Bantan, Bussum, North Holland | 52 | 16 | 5 | 12 | 09.05.1975 | male | + | + | + |
| NL77034 | toe pad | | | RMNH.AVES.77034 | The Netherlands | Huizen h. Fl. | 52 | 18 | 5 | 14 | 22.06.1977 | pull. | + | + | + |
| NL38833 | toe pad | | | ZMA.AVES.38833 | The Netherlands | Ermelo, Gelderland | 52 | 18 | 5 | 37 | 07.05.1995 | male | + | + | + |
| NL39269 | toe pad | | | ZMA.AVES.39269 | The Netherlands | Ermelo, Gelderland | 52 | 18 | 5 | 37 | 07.05.1995 | male | + | + | + |
| NL52592 | toe pad | | | ZMA.AVES.52592 | The Netherlands | Haren, Gelderland | 53 | 10 | 6 | 36 | 15.07.1995 | male | + | + | + |
| NL57320 | toe pad | | | ZMA.AVES.57320 | The Netherlands | Garderen, Wittenberg, Gelderland | 52 | 14 | 5 | 43 | 29.05.1999 | ? | + | + | + |
| 976284 | tissue | | | NRM976284 | Sweden | Åsele lappmark, Åsele, Norrstrand | 64 | 10 | 17 | 20 | 04.06.1997 | male | + | + | + |
| 976218 | tissue | | | NRM976218 | Sweden | Uppland, Björkö-Arholma, Simpnäs | 59 | 52 | 19 | 2 | 19.05.1997 | male | + | + | + |
| 986212 | tissue | | | NRM986212 | Sweden | Uppland, Tierp, Farsbo | 60 | 20 | 17 | 30 | 02.06.1998 | - | + | + | + |
| 996054 | tissue | | | NRM996054 | Sweden | Västerbotten, Umeå | 63 | 50 | 20 | 15 | 22.05.1998 | female | + | + | + |
| 986570 | tissue | | | NRM986570 | Sweden | Torne lappmark, Vittangi | 67 | 40 | 21 | 38 | 23.07.1997 | female | + | + | + |
| 996283 | tissue | | | NRM996283 | Sweden | Västerbotten, Umeå, Djänkerböle | 63 | 50 | 20 | 15 | 18.06.1999 | male | + | + | + |
| 6292 | tissue | | | NRM20026292 | Sweden | Lule lappmark, Jokkmokk, Kåbdalis, Luovare | 67 | 37 | 19 | 50 | 2002 | female | + | + | + |
| 6611 | tissue | | | NRM20016611 | Sweden | Småland, 7 km North of Emmaboda | 56 | 38 | 15 | 32 | 18.06.2001 | - | + | + | + |
| 6189 | tissue | | | NRM20056189 | Sweden | Uppland, Stockholm, Frescati | 59 | 21 | 18 | 4 | 24.05.2005 | male | + | + | - |
| 986427 | tissue | | | NRM986427 | Sweden | Öland, Ottenby | 56 | 14 | 16 | 25 | 11.05.1997 | male | + | + | + |
| 976132 | tissue | | | NRM976132 | Sweden | Blekinge, Ronneby, Tving | 56 | 12 | 15 | 17 | 17.07.1996 | male | + | + | + |
| 986133 | tissue | | | NRM986133 | Sweden | Uppland, Björkö-Arholma, Simpnäs | 59 | 52 | 19 | 2 | 29.04.1998 | male | + | + | + |
| 986188 | tissue | | | NRM986188 | Sweden | Uppland, Ekerö, Färingsö, Stenhamra | 59 | 23 | 17 | 38 | 11.05.1998 | male | + | + | + |
| 986424 | tissue | | | NRM986424 | Sweden | Öland, Ottenby | 56 | 14 | 16 | 25 | 04.05.1997 | female | + | + | + |
| 986425 | tissue | | | NRM986425 | Sweden | Öland, Ottenby | 56 | 14 | 16 | 25 | 11.05.1997 | female | + | + | + |
| 986426 | tissue | | | NRM986426 | Sweden | Öland, Ottenby | 56 | 14 | 16 | 25 | 11.05.1997 | male | + | + | + |
| 6670 | tissue | | | NRM20036670 | Sweden | Öland, Löttorp, Persnäs | 57 | 10 | 16 | 59 | 21.05.2003 | male | + | + | + |
| 6285 | tissue | | | NRM20006285 | Sweden | Blekinge, Karlskrona, Tving, Skärsjön | 56 | 11 | 15 | 39 | 25.05.2000 | male | + | + | + |
| 570365 | toe pad | | | NRM570365 | Sweden | Uppland, Uppsala | 59 | 51 | 17 | 37 | 15.05.1951 | male | + | + | + |
| 570356 | toe pad | | | NRM570356 | Sweden | Uppland, Uppsala | 59 | 51 | 17 | 37 | 28.05.1951 | female | + | + | + |
| 570360 | toe pad | | | NRM570360 | Sweden | Uppland, Uppsala | 59 | 51 | 17 | 37 | 03.06.1951 | - | + | + | + |
| 570354 | toe pad | | | NRM570345 | Sweden | Uppland, Uppsala | 59 | 51 | 17 | 37 | 11.05.1952 | male | + | + | + |
| 570366 | toe pad | | | NRM570366 | Sweden | Uppland, Uppsala | 59 | 51 | 17 | 37 | 26.06.1952 | male | + | + | + |
| 570362 | toe pad | | | NRM570362 | Sweden | Uppland, Uppsala | 59 | 51 | 17 | 37 | 11.05.1952 | male | + | - | + |
| 570355 | toe pad | | | NRM570355 | Sweden | Uppland, Uppsala | 59 | 51 | 17 | 37 | 12.05.1953 | male | + | + | + |
| 570353 | toe pad | | | NRM570353 | Sweden | Pite lappmark, Arvidsjaur | 65 | 35 | 19 | 10 | 15.08.1954 | female | + | + | + |
| 570357 | toe pad | | | NRM570357 | Sweden | Uppland, Tjärven near Norrtälje | 59 | 48 | 19 | 22 | 05.05.1954 | male | + | + | + |
| 570358 | toe pad | | | NRM570358 | Sweden | Uppland, Tjärven near Norrtälje | 59 | 48 | 19 | 22 | 05.05.1954 | female | + | + | + |
| 570361 | toe pad | | | NRM570361 | Sweden | Uppland, Dalby, Brunna | 58 | 13 | 11 | 39 | 12.06.1959 | male | + | + | + |
| 570359 | toe pad | | | NRM570359 | Sweden | Uppland, Norrtälje | 59 | 46 | 18 | 42 | 28.05.1960 | female | + | + | + |
| 570352 | toe pad | | | NRM570352 | Sweden | Uppland, Djursholm | 59 | 23 | 18 | 5 | 08.08.1961 | male | + | + | + |
| 570363 | toe pad | | | NRM570363 | Sweden | Småland, Byholma, Ränte | 60 | 6 | 18 | 49 | 19.05.1961 | female | + | + | + |
| 570351 | toe pad | | | NRM570351 | Sweden | Södermanland, Jönåker | 58 | 44 | 16 | 43 | 15.07.1962 | juv. | + | + | + |
| 570364 | toe pad | | | NRM570364 | Sweden | Västerbotten, Myrheden | 65 | 18 | 20 | 13 | 28.05.1966 | female | + | + | + |
| 670102 | toe pad | | | NRM670102 | Sweden | Södermanland, Enhöma, Horn (near Nyköping) | 58 | 45 | 17 | 0 | 08.06.1967 | - | + | + | + |
| 680100 | toe pad | | | NRM680100 | Sweden | Västmanland, Irsta | 59 | 36 | 16 | 42 | 29.05.1968 | male | + | + | + |
| 680099 | toe pad | | | NRM680099 | Sweden | Västmanland, Irsta | 59 | 36 | 16 | 42 | 29.05.1968 | male | + | + | + |
| 680151 | toe pad | | | NRM680151 | Sweden | Södermanland, Tyresö | 59 | 13 | 18 | 20 | 07.07.1968 | female | + | - | + |
|  |  | | |  |  |  |  |  |  |  |  |  |  |  |  |
| Samples sequenced but not included in final analyses   1. collected during the breeding period but outside Dutch forest breeding habitat 2. sampling of historical or recent population too small for analysis (n < 10) 3. further samples used for clock gene sequencing (not analyzed because of probable migrant status) | | | | | | | | | | | | | | | |
| NL9729 | | 1) | toe pad | RMNH.AVES.9729 | The Netherlands | Leiden |  |  |  |  | 22.05.1941 | male | + | + | + |
| NL20698 | | 1) | toe pad | RMNH.AVES.20698 | The Netherlands | Rinsumageest, Friesland |  |  |  |  | 15.05.1964 | male | + | + | + |
| NL2873 | | 1) | toe pad | RMNH.AVES.2873 | The Netherlands | Haamstede, lighthouse |  |  |  |  | 09.05.1913 | male | + | + | + |
| NL2875 | | 1) | toe pad | RMNH.AVES.2875 | The Netherlands | Wassenaar, dune |  |  |  |  | 10.05.1913 | male | + | + | - |
| NL2877 | | 1) | toe pad | RMNH.AVES.2877 | The Netherlands | Haamstede, lighthouse |  |  |  |  | 11.05.1913 | female | + | + | + |
| BMNH-1 | | 2) | toe pad | BMNH 1934.1.1.6256 | Portugal | Mauteigas |  |  |  |  | 07.05.1920 | male | + | - | - |
| BMNH-2 | | 2) | toe pad | BMNH 1934.1.1.6255 | Portugal | Mauteigas |  |  |  |  | 07.05.1920 | male | + | - | - |
| BMNH-3 | | 2) | toe pad | BMNH 1934.1.1.6250 | Spain | Segovia, San Ildefonso |  |  |  |  | 26.05.1927 | female | + | - | - |
| BMNH-4 | | 2) | toe pad | BMNH 1934.1.1.6249 | Spain | Segovia, San Ildefonso |  |  |  |  | 06.06.1931 | female | + | - | - |
| BMNH-5 | | 2) | toe pad | BMNH 1934.1.1.6253 | Spain | Segovia, San Ildefonso |  |  |  |  | 05.06.1931 | male | + | - | - |
| BMNH-6 | | 2) | toe pad | BMNH 1934.1.1.6252 | Spain | Segovia, San Ildefonso |  |  |  |  | 08.06.1931 | male | + | - | - |
| BMNH-8 | | 2) | toe pad | BMNH 1879.4.5.450 | Norway | South Norway |  |  |  |  | June 1866 | male | + | - | - |
| BMNH-9 | | 2) | toe pad | BMNH 1930.3.5.87 | Norway | Surendal |  |  |  |  | 15.06.1895 | male | + | - | - |
| BMNH-10 | | 2) | toe pad | BMNH 1899.8.9.61 | Norway | Sundalen |  |  |  |  | 06.07.1899 | male | + | - | - |
| BMNH-11 | | 2) | toe pad | BMNH 1955.3.350 | Norway | Sundalen |  |  |  |  | 19.06.1902 | male | + | - | - |
| BMNH-12 | | 2) | toe pad | BMNH 1947.4.156 | United Kingdom | Cley, Norfolk |  |  |  |  | May 1911 | male | + | - | - |
| BMNH-13 | | 2) | toe pad | BMNH 1953.76.183 | United Kingdom | Cley, Norfolk |  |  |  |  | 07.05.1936 | male | + | - | - |
| BMNH-14 | | 2) | toe pad | BMNH 1953.76.182 | United Kingdom | Cley, Norfolk |  |  |  |  | 13.05.1936 | male | + | - | - |
| BMNH-15 | | 2) | toe pad | BMNH 1953.76.184 | United Kingdom | Salthouse, Norfolk |  |  |  |  | 13.05.1936 | male | + | - | - |
| BMNH-16 | | 2) | toe pad | BMNH 1934.1.1.6037 | United Kingdom | Garth, Breconshire |  |  |  |  | 24.07.1932 | female (juv) | + | - | - |
| BMNH-17 | | 2) | toe pad | BMNH 1934.1.1.223 | United Kingdom | Garth, Breconshire |  |  |  |  | 03.08.1932 | male (juv) | + | - | - |
| BMNH-18 | | 2) | toe pad | BMNH 1934.1.1.224 | United Kingdom | Garth, Breconshire |  |  |  |  | 05.08.1032 | juv | + | - | - |
| MTD794 | | 2) | toe pad | MTD C41926 | Russia | Smolensk, Schujatschi |  |  |  |  | 05.05.1943 | male | + | - | - |
| MTD795 | | 2) | toe pad | MTD C41927 | Russia | Smolensk, Schujatschi |  |  |  |  | 30.05.1943 | male | + | - | - |
| MTD797 | | 2) | toe pad | MTD C41928 | Russia | Smolensk, Orscha |  |  |  |  | 27.04.1944 | male | + | - | - |
| MTD 796 | | 2) | toe pad | MTD C37057 | Ukraine | Oblast Tschernihiw, Oster |  |  |  |  | 26.04.1943 | male | + | - | - |
| MAR241 | | 3) | blood | - | Czech Republic | Bohemia, Šumava |  |  |  |  | 09.08.1995 | female | + | - | + |
| MAR498 | | 3) | blood | - | Germany | France, Gironde, Montavilet |  |  |  |  | 25.08.1998 | male | + | - | + |
| MAR3013 | | 3) | blood | - | Germany | Lake Constance, Mettnau |  |  |  |  | 20.09.2001 | - | + | - | + |
| MAR3090 | | 3) | blood | - | Germany | Lake Constance, Mettnau |  |  |  |  | 27.08.2001 | male | + | - | + |
| MAR3862 | | 3) | blood | - | Germany | Lower Saxony, Pollhagen |  |  |  |  | 01.09.2003 | male | + | - | + |
| MAR4976 | | 3) | blood | - | Germany | Rhineland-Palatinate, near Eich |  |  |  |  | 13.08.2005 | - | + | - | + |
| MAR4998 | | 3) | blood | - | Germany | Rhineland-Palatinate, near Eich |  |  |  |  | 27.08.2005 | - | + | - | + |
| MAR5051 | | 3) | blood | - | Italy | Alto Adige, Völser Aicha |  |  |  |  | 25.08.2005 | - | + | - | + |
| MAR5058 | | 3) | blood | - | Italy | Alto Adige, Völser Aicha |  |  |  |  | 26.08.2005 | - | + | - | + |
| MAR5059 | | 3) | blood | - | Italy | Alto Adige, Völser Aicha |  |  |  |  | 26.08.2005 | - | + | - | + |
| MAR5063 | | 3) | blood | - | Italy | Alto Adige, Völser Aicha |  |  |  |  | 26.08.2005 | - | + | - | + |
| MAR5654 | | 2) | blood | - | Germany | Rhineland-Palatinate, near Eich |  |  |  |  | 17.06.2006 | - | + | - | + |
| MAR7315 | | 3) | blood | - | Germany | Black Forest, Grafenhausen |  |  |  |  | 18.09.2008 | - | + | - | + |
