# Supplementary material for: Differentiation in neutral genes and a candidate gene in the pied flycatcher: using biological archives to track global climate change
Source: Ecol Evol. 2013 Nov 1;3(14):4799–814. doi: 10.1002/ece3.855 (PMC3867912; doi:10.1002/ece3.855)
Supplement: Supplementary file 2 [file ece30003-4799-SD2.doc]

**Table S2.** Microsatellite loci (according to Leder *et al.* 2008), forward and reverse primer sequences (5´ 3´), labelling dye and final primer concentrations used for the multiplex PCR-reaction.

| **Locus** | **forward sequence** | **reverse sequence** | **dye** | **concentration** |
| --- | --- | --- | --- | --- |
| Fhy216 | GGGTGCTGCTCAAGGACTTC | GTTTAGCCAACACCATCGTTCTG | Alexa647 | 0.4 µM |
| Fhy407 | AAAGTTAGCCTATGTCTACCAGA | GTTTAGCTCTTCCCAGATTCTAAG | Alexa647 | 0.5 µM |
| Fhy401 | TCAAATATTAATTGGTTACACTT | GTTTCTCTTAAACTAACAACTTGCTAA | Alexa647 | 0.2 µM |
| Fhy326 | TCAGATTGTGCTGGTAATG | GTTTCCTAAAGGGAGTATAAGCAAC | Alexa647 | 0.2 µM |
| Fhy361 | ATGCTGAGCCAAAGTCTGTC | GTTTATCAGGGAGCTGAGACGAG | Alexa647 | 0.2 µM |
| Fhy223 | TTCTCCTGGTCCTTAGCTTA | GTTTCCATCTGCTTCTCTATCCCC | IRD700 | 0.15 µM |
| Fhy235 | AATGAGTGTAACAGTAGGG | GTTTGTCTATGTAAACCTAAAGC | IRD700 | 0.15 µM |
| Fhy329 | AAACTTGAGCTGAGGTTCA | GTTTAAAGCTACATCGCAGTG | IRD700 | 0.25 µM |
| Fhy452 | ATATCCAATAGCCGCAAACT | GTTTGGATGCAGATTATTAGCCTT | IRD700 | 0.3 µM |
| Fhy339 | ATGAAGGAGGTGATGGTAAAT | GTTTAAACACAGCACAGCATAACAT | IRD700 | 0.3 µM |
