# Supplementary material for: Differentiation in neutral genes and a candidate gene in the pied flycatcher: using biological archives to track global climate change
Source: Ecol Evol. 2013 Nov 1;3(14):4799–814. doi: 10.1002/ece3.855 (PMC3867912; doi:10.1002/ece3.855)
Supplement: Supplementary file 3 [file ece30003-4799-SD3.doc]

**Table S3.** Pairwise F_st_ comparisons between all populations: A) based on control region, B) based on microsatellite loci and C) based on *Clock*-gene loci

**A) control region**

|  |  | Finland | Germany | | Norway | Spain | Sweden | | The Netherlands | |
| --- | --- | --- | --- | --- | --- | --- | --- | --- | --- | --- |
|  |  | recent | recent | historic | recent | recent | recent | historic | recent | historic |
| Finland | recent | - |  |  |  |  |  |  |  |  |
| Germany | recent | 0.097* | - |  |  |  |  |  |  |  |
|  | historic | 0.102* | 0,000 | - |  |  |  |  |  |  |
| Norway | recent | 0.032 | 0.045* | 0.041* | - |  |  |  |  |  |
| Spain | recent | 0.540* | 0.529* | 0.524* | 0.497* | - |  |  |  |  |
| Sweden | recent | 0.000 | 0.081* | 0.060* | 0.000 | 0.527* | - |  |  |  |
|  | historic | 0.000 | 0.051 | 0.060* | 0.000 | 0.502* | 0,000 | - |  |  |
| The | recent | 0.141* | 0.000 | 0.013 | 0.070* | 0.520* | 0.110* | 0.088* | - |  |
| Netherlands | historic | 0.313* | 0.059* | 0.073* | 0.201* | 0.578* | 0.270* | 0.234* | 0.033* | - |

B) microsatellites

|  |  | Finland | Germany | | Norway | Spain | Sweden | | The Netherlands | |
| --- | --- | --- | --- | --- | --- | --- | --- | --- | --- | --- |
|  |  | recent | recent | historic | recent | recent | recent | historic | recent | historic |
| Finland | recent | - |  |  |  |  |  |  |  |  |
| Germany | recent | 0.011* | - |  |  |  |  |  |  |  |
|  | historic | 0.018* | 0.008* | - |  |  |  |  |  |  |
| Norway | recent | 0.003 | 0,000 | 0.002 | - |  |  |  |  |  |
| Spain | recent | 0.041* | 0.032* | 0.031* | 0.029* | - |  |  |  |  |
| Sweden | recent | 0.026* | 0.007 | 0.011* | 0.011 | 0.026* | - |  |  |  |
|  | historic | 0.029* | 0.012* | 0.011* | 0.010 | 0.043* | 0.006 | - |  |  |
| The | recent | 0.020* | 0.015* | 0.016* | 0.011* | 0.040* | 0.017* | 0.016* | - |  |
| Netherlands | historic | 0.029* | 0.021* | 0.015* | 0.017* | 0.048* | 0.018* | 0.016* | 0.006 | - |

**C) clock gene**

|  |  | Finland | Germany | | Norway | Spain | Sweden | | The Netherlands | |
| --- | --- | --- | --- | --- | --- | --- | --- | --- | --- | --- |
|  |  | recent | recent | historic | recent | recent | recent | historic | recent | historic |
| Finland | recent | - |  |  |  |  |  |  |  |  |
| Germany | recent | 0.000 | - |  |  |  |  |  |  |  |
|  | historic | 0.000 | 0.000 | - |  |  |  |  |  |  |
| Norway | recent | 0.000 | 0.000 | 0.000 | - |  |  |  |  |  |
| Spain | recent | 0.000 | 0.000 | 0.000 | 0.008 | - |  |  |  |  |
| Sweden | recent | 0.000 | 0.000 | 0.000 | 0.000 | 0.008 | - |  |  |  |
|  | historic | 0.000 | 0.000 | 0.000 | 0.000 | 0.000 | 0.006 | - |  |  |
| The | recent | 0.000 | 0.000 | 0.000 | 0.000 | 0.017* | 0.000 | 0.001 | - |  |
| Netherlands | historic | 0.000 | 0.000 | 0.012 | 0.000 | 0.000 | 0.000 | 0.000 | 0.000 | - |

* indicates significance (p <0.05).
