# Supplementary material for: Differentiation in neutral genes and a candidate gene in the pied flycatcher: using biological archives to track global climate change
Source: Ecol Evol. 2013 Nov 1;3(14):4799–814. doi: 10.1002/ece3.855 (PMC3867912; doi:10.1002/ece3.855)
Supplement: Supplementary file 4 [file ece30003-4799-SD4.doc]

**Table S4.** Mean number of *Clock*-gene Q-repeats in male and female pied flycatchers (*F. hypoleuca*) found in different populations over space and time (both alleles added for each individual); n.a. = not analyzed separately for sexes due to a large number of nestling specimens without sex determination.

|  | Finland | Germany historic | Germany recent | Norway | Spain | Sweden recent | Sweden historic | The  Netherlands  recent | The  Netherlands historic | mean |
| --- | --- | --- | --- | --- | --- | --- | --- | --- | --- | --- |
| male | 24.67 | 24.29 | n.a. | 24.36 | 23.71 | 24.20 | 24.10 | 24.36 | 24.00 | 24.21 |
| female | 23.71 | 23.78 | n.a. | 24.20 | 23.92 | 24.40 | 23.71 | 23.94 | 23.75 | 23.93 |
| mean | 24.19 | 24.04 | 24.00 | 24.28 | 23.82 | 24.30 | 23.91 | 24.15 | 23.88 |  |
