# Supplementary figures and images for: Differentiation in neutral genes and a candidate gene in the pied flycatcher: using biological archives to track global climate change
Source: Ecol Evol. 2013 Nov 1;3(14):4799–814. doi: 10.1002/ece3.855 (PMC3867912; doi:10.1002/ece3.855)

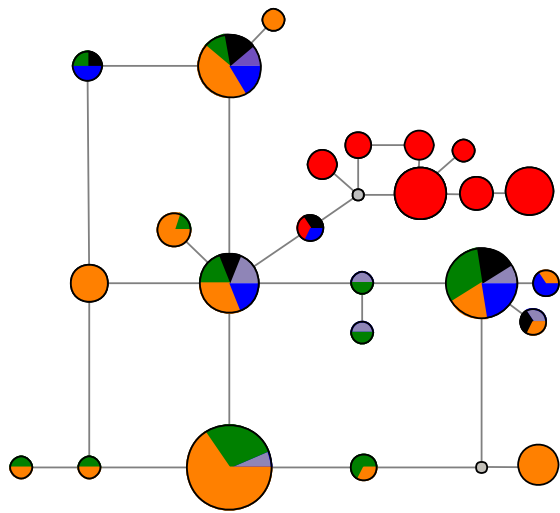

- Finland
- Germany
- Norway
- Spain
- Sweden
- The Netherlands

Supplement: Supplementary file 5 [file ece30003-4799-SD5.pdf]

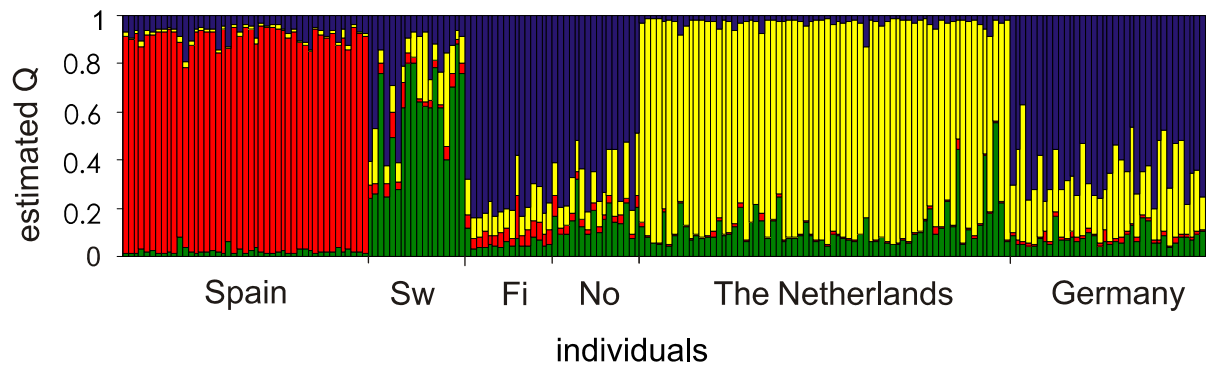

Supplement: Supplementary file 6 [file ece30003-4799-SD6.pdf]
